# Systematic Review and Meta-analysis of Candidate Gene Association Studies of Lower Urinary Tract Symptoms in Men^☆^

**DOI:** 10.1016/j.eururo.2014.01.007

**Published:** 2014-10

**Authors:** Rufus Cartwright, Altaf Mangera, Kari A.O. Tikkinen, Prabhakar Rajan, Jori Pesonen, Anna C. Kirby, Ganesh Thiagamoorthy, Chris Ambrose, Juan Gonzalez-Maffe, Phillip R. Bennett, Tom Palmer, Andrew Walley, Marjo-Riitta Järvelin, Vik Khullar, Chris Chapple

**Affiliations:** a Department of Epidemiology and Biostatistics and Department of Urogynaecology, Imperial College London, London, UK; b Department of Urology Research, University of Sheffield, Sheffield, UK; c Department of Urology, Helsinki University Central Hospital and University of Helsinki, Helsinki, Finland, and Department of Clinical Epidemiology and Biostatistics, McMaster University, Hamilton, ON, Canada; d Beatson Institute for Cancer Research, University of Glasgow, Glasgow, UK; e Department of Urology, Tampere University Hospital, and School of Medicine, University of Tampere, Tampere, Finland; f Department of Reproductive Medicine, University of California, San Diego, San Diego, CA, USA, and Department of Obstetrics and Gynecology, Kaiser Permanente, San Diego, CA, USA; g Department of Urogynaecology, King's College London, London, UK; h University College London Medical School, London, UK; i Clinical Trials Unit, Imperial College London, London, UK; j Institute for Reproductive and Developmental Biology, Imperial College London, London, UK; k Division of Health Sciences, Warwick Medical School, University of Warwick, Coventry, UK; l Section of Genomic Medicine, National Heart and Lung Institute and Department of Genomics of Common Disease, School of Public Health, Imperial College London, London, UK; m Department of Epidemiology and Biostatistics, MRC Health Protection Agency (HPA), Centre for Environment and Health, School of Public Health, Imperial College, London, UK, Institute of Health Sciences and Biocentre, Oulu, University of Oulu, Finland, Unit of Primary Care, Oulu University, Hospital, Oulu, Finland and Department of Children and Young People and Families, National Institute for Health and Welfare, Oulu, Finland; n Department of Urogynaecology, Imperial College London, London, UK; o Department of Urology, Sheffield Teaching Hospitals NHS Foundation Trust, Sheffield, UK

**Keywords:** Benign prostatic hyperplasia, BPH, Genetics, Genomics, Lower urinary tract symptoms, LUTS, Incontinence, male, Overactive bladder

## Abstract

**Context:**

Although family studies have shown that male lower urinary tract symptoms (LUTS) are highly heritable, no systematic review exists of genetic polymorphisms tested for association with LUTS.

**Objective:**

To systematically review and meta-analyze studies assessing candidate polymorphisms/genes tested for an association with LUTS, and to assess the strength, consistency, and potential for bias among pooled associations.

**Evidence acquisition:**

A systematic search of the PubMed and HuGE databases as well as abstracts of major urologic meetings was performed through to January 2013. Case-control studies reporting genetic associations in men with LUTS were included. Reviewers independently and in duplicate screened titles, abstracts, and full texts to determine eligibility, abstracted data, and assessed the credibility of pooled associations according to the interim Venice criteria. Authors were contacted for clarifications if needed. Meta-analyses were performed for variants assessed in more than two studies.

**Evidence synthesis:**

We identified 74 eligible studies containing data on 70 different genes. A total of 35 meta-analyses were performed with statistical significance in five (*ACE, ELAC2, GSTM1, TERT*, and *VDR*). The heterogeneity was high in three of these meta-analyses. The rs731236 variant of the vitamin D receptor had a protective effect for LUTS (odds ratio: 0.64; 95% confidence interval, 0.49–0.83) with moderate heterogeneity (I^2^ = 27.2%). No evidence for publication bias was identified. Limitations include wide-ranging phenotype definitions for LUTS and limited power in most meta-analyses to detect smaller effect sizes.

**Conclusions:**

Few putative genetic risk variants have been reliably replicated across populations. We found consistent evidence of a reduced risk of LUTS associated with the common rs731236 variant of the vitamin D receptor gene in our meta-analyses.

**Patient summary:**

Combining the results from all previous studies of genetic variants that may cause urinary symptoms in men, we found significant variants in five genes. Only one, a variant of the vitamin D receptor, was consistently protective across different populations.

## Take Home Message

Despite >70 publications, the evidence for associations between any specific genetic polymorphism and male lower urinary tract symptoms (LUTS) remains weak. In pooled analyses, only the rs731236 variant of the vitamin D receptor has moderate epidemiologic credibility as a risk factor for LUTS.

## 1 Introduction

Lower urinary tract symptoms (LUTS) in men are categorized into storage symptoms (increased daytime urinary frequency, nocturia, urgency, and incontinence), voiding symptoms (slow stream, splitting or spraying, intermittent stream, hesitancy, straining, and terminal dribble), and postmicturition symptoms (feeling of incomplete emptying and postmicturition dribble) [1], [2]. LUTS are highly prevalent and often bothersome. They are strongly associated with both age and obesity [3], [4], [5], which is therefore likely to increase future associated costs and burden.

Particularly when considering older men, a variety of terms have been used historically to describe LUTS including symptomatic benign prostatic hyperplasia (BPH), symptomatic benign prostatic enlargement (BPE), or symptomatic bladder outlet obstruction [6]. However, only a minority of men with histologic evidence of BPH develop significant bothersome LUTS, and among men presenting with LUTS, only a minority have obstruction [6]. With increasing focus on medical therapies targeting either the bladder or prostate [7], the non–organ-specific term LUTS has therefore been recommended, emphasizing the multiple potential etiologies for these symptoms.

There is substantial evidence of familial aggregation of male LUTS. Early reports identified very large excess risks for the surgical treatment of LUTS among men with so-called familial BPH [8]. However, subsequent work has suggested more modest familial risks for the symptoms themselves [9], [10]. In the Olmsted County study, having either a father or a brother with a history of diagnosed BPE was associated with an odds ratio (OR) of 1.5 (95% confidence interval [CI], 1.1–1.7) for moderate to severe LUTS at baseline [9]. In the Krimpen study, reporting any first- or second-degree relative with a diagnosis of prostate cancer was associated with a hazard ratio of 1.7 (95% CI, 1.1–2.5) for incident LUTS over a median of 6.5 yr of follow-up [10]. Such risks seem to be cumulative, with two or more affected relatives conferring greater risk [11].

Twin studies provide estimates of heritability that are less confounded by environmental or lifestyle factors that may be shared within families. In a study of 256 twin pairs enrolled in the US military, heritability was estimated at 49% using a case definition corresponding to diagnosis and/or treatment for BPH [12]. In a population-based study of 83 twin pairs, the heritability of the American Urological Association Symptom Index (AUA-SI) was estimated at 39% overall, but with a higher heritability of 83% for men >50 yr of age [13]. In a further population-based study of 3446 elderly male twins, heritability of moderate to severe LUTS (again assessed using the AUA-SI) was estimated at 72% [14]. Taken together, these twin studies suggest similar heritability as for many complex diseases for which the genetic architecture is well understood, including prostate cancer, where heritability has been estimated at between 42% [15] and 58% [16].

Many of the studies available for this review aimed primarily to explore the molecular genetics of prostate cancer rather than LUTS, but they included men with and without LUTS as separate subgroups of controls. It remains unclear whether LUTS or BPH might be risk factors for prostate cancer. There is conflicting data regarding any association of a diagnosis of LUTS/BPH with a subsequent diagnosis of prostate cancer [17], [18], [19], [20]. Evidence of a consequent increase in high-risk cancers or prostate cancer mortality is also mixed. Those studies that have suggested a positive association may be unable to exclude detection bias and unmeasured confounding from shared environmental or genetic risk factors.

With pharmaceutical options for the prevention of prostate cancer and LUTS [21], and an expanding array of conservative options for managing LUTS, clinical risk stratification may become more relevant than ever. Robustly replicated genetic variants associated with LUTS would provide useful information in assessing both prognosis and potentially treatment response. Equally importantly, new insights into the molecular genetics of LUTS could help explain the underlying pathogenesis and also offer future routes toward new drug targets.

The aim of this systematic review was to assess which candidate polymorphisms and/or candidate genes had been tested for an association with LUTS in men, and to assess the strength, consistency, and potential for bias among pooled associations.

## 2 Evidence acquisition

### 2.1 Eligibility criteria

The review protocol was prospectively registered (PROSPERO 2011: CRD42012001985). We prespecified inclusion of both case-control and cross-sectional designs, with both population-based samples and other sampling methods. We included association studies testing for any genetic polymorphism at the nucleotide level including single nucleotide polymorphisms (SNPs), deletions, duplications, and copy-number variants but excluded larger microscopic variants at the karyotype level.

For LUTS, there are no gold standard diagnostic methods because these are largely symptomatic diagnoses. We therefore expected to accept case definitions or criteria for LUTS as specified within each study, recognizing there would be heterogeneity in definitions across studies. We planned to include case definitions based on validated symptom questionnaires, clinical evaluation, or urodynamics. After conducting initial searches, we expanded this to case definitions based on care seeking, including the use of relevant medications (eg, α-blockers or anticholinergics) or a history of relevant surgery including transurethral resection of the prostate. We excluded studies using solely histologic BPH or clinical BPE case definitions where LUTS were not an inclusion criterion, for example studies based on samples of asymptomatic men undergoing prostate cancer screening. We considered the population of interest as men ≥18 yr of age.

### 2.2 Search strategy

We combined searches from PubMed, HuGE Navigator, and an extensive selection of urologic conference reports. We searched PubMed up to January 2013 without language restrictions, using a combination of genetic and phenotype keywords and Medical Subject Headings (MeSH) terms: *(polymorphism OR SNP OR CNV OR “copy number variation” OR mutation OR genetic OR chromosome OR VNTR OR InDel OR microsatellite) AND (“benign prostatic enlargement” OR BPE OR “benign prostatic hyperplasia” OR “bladder outflow obstruction” OR BPH OR nocturia OR LUTS OR incontinence OR urgency OR “overactive bladder” OR “Lower Urinary Tract Symptoms”[Mesh] OR “Urinary Incontinence”[MeSH] OR “enuresis”[Mesh]) NOT mitral NOT carcinoma[Title] NOT cancer[Title] NOT (animals[mh] NOT humans[mh])*

We searched HuGE Navigator, also through to January 2013, using the following phenotype indexing terms: (“prostatic diseases” OR “prostatic hyperplasia” OR “urination disorders” OR “nocturia” OR “urinary incontinence” OR “urinary bladder, overactive”).

In addition we searched conference abstracts for annual meetings of the American Urological Association, European Association of Urology, International Urogynecological Association, and International Continence Society from 2005 to 2012.

### 2.3 Screening and data extraction

We developed standardized data forms, and conducted pilot screening and data extraction training exercises to achieve a high level of consensus between reviewers. All screening and data extraction was performed independently and in duplicate by methodologically trained reviewers. Reviewers screened study reports by first screening titles and abstracts to select papers for full-text assessment and then screening full-text papers to confirm eligibility of the articles. Screening discrepancies were resolved by discussion and adjudication. We hand-searched reference lists of all included articles, applying the same standardized screening process. When more than one published or unpublished report was identified for the same association in the same study population, we included the paper or abstract with the largest sample size.

We contacted study authors by e-mail for clarifications, additional information about methodology, and for additional subgroup analyses where necessary. Data extracted included information on the setting for each study, details of the sampling strategy and sampled populations (age, ethnic/racial composition, and body mass index), the overall sample size and proportion genotyped, the outcome assessments used and phenotypic definitions, the genotyping method used, and the genotyping quality control methods applied. Where possible we extracted or requested from authors full genotype frequencies among both cases and controls.

### 2.4 Statistical analysis and risk of bias assessments

For polymorphisms assessed in at least two studies for the same phenotype, we conducted meta-analyses using the “metan” package (Stata v.12.1; StataCorp, College Station, TX, USA). For meta-analyses with only two studies, or for three or more studies and low heterogeneity, we used fixed effects models but otherwise used random effects models. In the absence of a clear rationale supporting any specific model of inheritance, we used the allelic association test, corresponding to codominant modes of inheritance for all polymorphisms. We assessed the credibility of pooled associations using the interim Venice criteria [22] that rates pooled associations as weakly, moderately, or strongly credible (see summary in Supplemental Fig. 17). We used the Cochran *Q* test and the I^2^ statistic as measures of between-study heterogeneity. We retested for departure from the Hardy-Weinberg equilibrium among controls. We assessed the risk of bias in phenotype definitions, genotyping, and population stratification. We used the Harbord [23] and Egger [24] tests of funnel plot asymmetry to investigate possible reporting biases. Reporting of the review complies with recommendations both of the HuGE Handbook [25] and the Preferred Reporting Items for Systematic Reviews and Meta-analysis statement [26].

## 3 Evidence synthesis

**Fig. 1:**
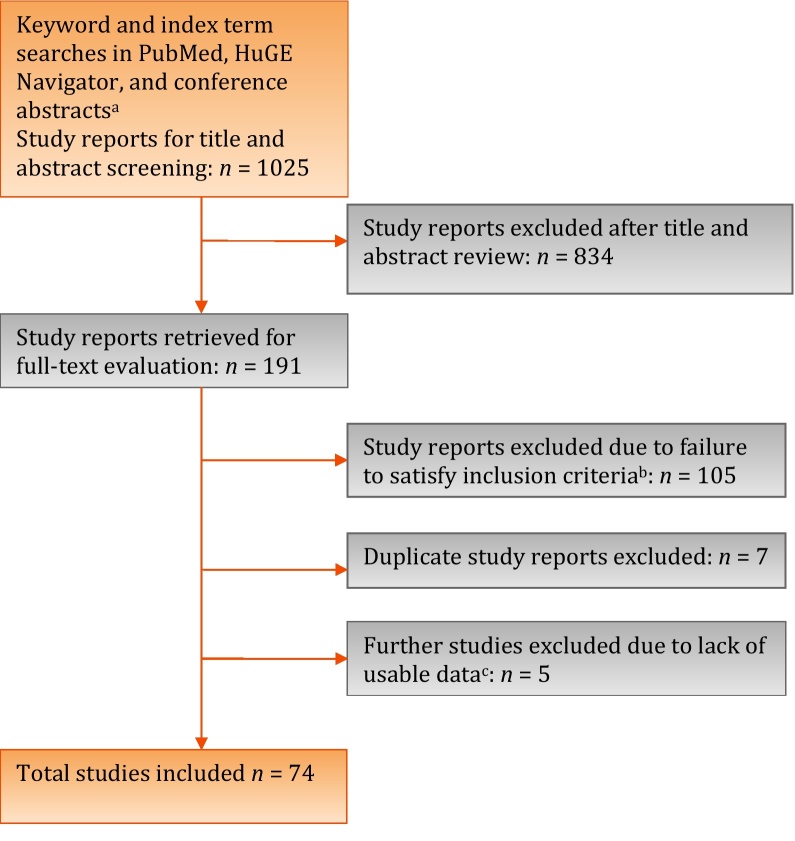
Flowchart outlining the literature search and article evaluation process. ^a^ International Continence Society, International Urogynecological Association, American Urological Association, and European Association of Urology abstracts 2005–2012, using search interfaces at http://www.icsoffice.org/Abstracts/, http://www.sciencedirect.com/science/journal/15699056, http://www.jurology.com/supplements, and/or full-text search of abstract book PDFs. ^b^ Includes studies enrolling only women (*n* = 32), only children (*n* = 2), reviews or letters (*n* = 12), inapplicable phenotypes such as prostate cancer/prostate-specific antigen/benign prostatic enlargement/histologic benign prostatic hyperplasia (*n* = 47), cohort study reports (*n* = 5), and other study designs including pharmacogenetic studies, gene expression studies, and polymorphic protein studies (*n* = 4). ^c^ Authors contacted by e-mail for additional data from 37 studies.

We screened 1025 abstracts and retrieved 191 full texts (Fig. 1). A total of 74 study reports provided data (Table 1) regarding polymorphisms in or near 70 different genes (Supplemental Table 1). We found no relevant genome-wide association studies (GWAS) for male LUTS, with all included studies using a candidate gene approach. Most research interest has focused on variation in genes implicated in steroid metabolic processes, inflammatory response, and cytokine activity (Supplemental Table 2). With many studies prioritizing prostate cancer candidate genetic variation for investigation, there was also a significant overrepresentation of genes implicated in the regulation of cell proliferation and apoptosis. Only two studies considered specific LUTS [27], [28], with all other studies addressing a composite definition of male LUTS suggestive of BPH.

**Table 1:** Included studies

Study	Country	Descent/ethnicity/race*	Gene symbols(s)	Polymorphism(s) dbSNP ID or other identifier	LUTS case definition 1: Validated questionnaire 2: BOO surgery 3: Nonvalidated assessment 4: Care seeking	Additional assessment(s) 1: DRE 2: PSA 3: TRUS 4: Histology 5: Flow studies
Ashtiani et al. [58]	Iran	Iranian	*AR* *GSTM1* *GSTT1*	CAG repeat Null genotype Null genotype	3, 4	2, 3, 4
Berhane et al. [59]	India	North Indian	*ERCC5 XRCC1*	rs17655 rs25487	3	4
Bid et al. [31]	India	Indian	*ACE*	rs4340	1, 4	1, 2, 3
Biolchi et al. [60]	Brazil	>80% white	*AR*	CAG repeat	3, 4	1, 3, 4
Biolchi et al. [61]	Brazil	>80% white	*AR*	GGC repeat	3, 4	1, 3, 4
Bousema et al. [46]	Netherlands	Dutch	*VDR*	rs731236	3, 4	1, 2, 5
Chaimuangraj et al. [49]	Thailand	Thai	*VDR*	rs731236 rs1544410 rs7975232	2, 3, 4	2, 4
Choubey et al. [38]	India	Indian	*GSTM1* *GSTT1*	Null genotype Null genotype	1, 4	2, 3
Faria et al. [62]	Brazil	Brazilian	*TGFB1*	rs1800471 rs1800470	2	4
Giovannucci et al. [52]	USA	Mixed US	*AR*	CAG repeat	1, 2	1, 4
Giovannucci et al. [53]	USA	Mixed US	*AR*	CAG repeat	2	4
Gudmundsson et al. [41]	Iceland	Icelandic	*TERT* *MSMB* *FGFR2* *TBX3* *HNF1B* *KLK3*	rs2736098† rs401681 rs10993994 rs10788160 rs11067228 rs4430796 rs17632542 rs2735839	2, 4	
Gunes et al. [63]	Turkey	Turkish	*KLK3* *CYP17A1*	rs266882 rs743572	2, 3, 4	4
Gupta et al. [64]	India	Indian	*ESR1*	rs9340799 rs2234693‡	4	2, 4
Habuchi et al. [65]	Japan	Japanese	*CYP17A1*	rs743572	3, 4	1, 2
Habuchi et al. [47]	Japan	Japanese	*VDR*	rs731236 rs1544410 rs7975232	3, 4	1, 2
Hamasaki et al. [48]	Japan	Japanese	*VDR*	rs731236	3, 4	1, 2, 3
Helfland et al. [42]	USA	White	*RP11-382A18.1* *TERT* *MSMB* *FGFR2* *TBX3* *HNF1B* *KLK3*	rs1447295† rs6983267 rs2736098 rs401681 rs10993994 rs10788160 rs11067228 rs4430796 rs17632542 rs2735839	1, 4	2, 5
Ho et al. [66]	Scotland	White	*FGFR4*	rs351855	4	
Huang et al. [67]	Taiwan	Taiwanese	*VDR*	rs10735810	1, 3, 4	1, 2, 3
Huang et al. [68]	Taiwan	Taiwanese	*TP53* *CDKN1A*	rs1042522 rs1801270	1, 3, 4	1, 2, 3
Izmirli et al. [35]	Turkey	Southern Turkish	*SRD5A2* *ELAC2*	rs523349 rs9282858 rs4792311 rs5030739	4	
Jerónimo et al. [69]	Portugal	Unclear	*GSTP1*	rs1695	2, 4	4
Kamoto et al. [70]	Japan	Japanese	*CDH1*	rs16260	3, 4	1, 2
Kesarwani and Mittal [71]	India	North Indian	*IL1B* *TNF* *IFNG* *IL1RN* *IL4* *IL6* *Il10* *TGFB1*	rs16944 rs1800629 rs1799964 rs1800630 rs1799724 rs2430561 rs2234663 rs2234664 rs2069840 rs1800896 rs1800871 rs1800470	1, 4	1, 2, 4
Konwar et al. [72]	India	North Indian	*IL4* *IL1RN*	rs2234664 rs2234663	1, 4	1, 2, 3, 4
Konwar et al. [73]	India	North Indian	*GSTT1* *GSTM1* *GSTP1*	Null genotype Null genotype rs1695	1, 4	1, 2, 3, 5
Kristal et al. [74]	USA	>90% white	*AR*	CAG repeat	1	1, 2
Kumar et al. [75]	India	White Aryan	*GSTT1* *GSTM1*	Null genotype Null genotype	3, 4	1, 2, 3, 4
Kumazawa et al. [76]	Japan	Japanese	*CYP11A1*	(TTTTA)n	3, 4	1, 2
Li et al. [77]	Sweden/Japan	Swedish/Japanese	*AR*	CAG repeat	3, 4	1, 2, 3
Li et al. [78]	Japan	Japanese	*SRD5A2*	rs523349 rs9282858	3, 4	1, 2, 3
Li et al. [79]	Japan	Japanese	*TGFB1*	rs1800470	3, 4	1, 2, 3
Licastro et al. [80]	Italy	Italian	*SERPINA3*	rs1884082	3, 4	1, 2, 3
Ma et al. [81]	Japan	Japanese	*FGFR4*	rs2011077 rs351855	1, 3, 4	1, 2, 3
Madigan et al. [82]	China	Chinese	*CYP17*	rs743572	2, 4	1, 2, 4
Manchanda et al. [50]	India	North Indian	*VDR*	rs731236 rs1544410 rs10735810	1, 4	1, 2, 3
Mitsumori et al. [83]	Japan	Japanese	*AR*	CAG repeat	2, 4	4
Mittal et al. [84]	India	North Indian	*IL1RN*	rs2234663	4	2, 3, 4
Mittal et al. [85]	India	North Indian	*GSTM1* *GSTT1* *GSTM3*	Null genotype Null genotype rs1799735	1, 4	1, 2, 4
Mononen et al. [86]	Finland	Finnish	*AR*	CAG repeat	4	3, 5
Mononen et al. [87]	Finland	Finnish	*SRD5A2*	rs9282858	4	3, 5
Narita et al. [88]	Japan	Japanese	*LPL*	rs254 rs316 rs328	3, 4	1, 2, 3
Nikolić et al. [89]	Serbia	Serbian	*Intergenic*	rs3787016	3, 4	1, 2, 3
Omrani et al. [90]	Iran	Iranian	*TGFB1*	rs1800470	4	1, 2, 4
Omrani et al. [91]	Iran	Iranian	*IL10* *IFNG* *TNF*	rs1800896 rs2430561 rs1800629	4	1, 2
Rajender et al. [92]	India	South Indian	*SRD5A2*	rs523349 rs9282858 TA(n) repeat	4	1, 2
Rökman et al. [33]	Finland	Finnish	*ELAC2*	rs5030739 rs4792311 rs119484087	3, 4	2, 3
Rökman et al. [93]	Finland	Finnish	*RNASEL*	rs486907 rs74315364 rs627928 rs145787003	3,4	2, 3, 5
Safarinejad et al. [94]	Iran	White	*IGFBP3*	rs2854744	1, 4	1, 2, 3, 5
Salam et al. [95]	USA	Mixed US	*SRD5A2*	rs523349 rs9282858 TA(n) repeat	1	1, 2
Schwanke et al. [28]	Brazil	Mixed Brazilian	*HTR2A*	rs6313	3	
Seppälä et al. [96]	Finland	Finnish	*KLF6*	rs3750861	3, 4	1, 2, 3, 5
Shibata et al. [97]	Japan	Japanese	*ADRA1A*	rs1048101	3, 4	1
Sierra Diaz et al. [30]	Mexico	Mexican	*ACE* *AGTR1*	rs4340 rs5186	2, 4	4
Sobti et al. [98]	USA	North Indian	*ESR1* *SRD5A2* *KLK3* *CYP17*	rs2234693‡ TA(n) repeat rs266882 rs743572	1, 4	2, 4
Sobti et al. [36]	India	North Indian	*ELAC2* *SERPINA1*	rs4792311 rs5030739 rs28929474 rs17580	1, 4	2, 4
Steiner et al. [99]	Germany	White	*NQO1*	rs1800566	2	4
Takeda et al. [27]	Japan	Japanese	*ADRA1A* *ADRB3*	rs1048101 rs4994	3, 4	
Takahashi et al. [100]	Japan	Japanese	*ELAC2*	rs4792311 rs5030739 rs78105154	4	
Tanaka et al. [101]	Japan	Japanese	*COMT*	rs4633 rs4680 rs6267	2,4	2, 4
Tanaka et al. [102]	Japan	Japanese	*MLH1*	rs28930073 rs1799977 rs63750447 p.Ala723Asp	2,4	2, 4
Teitsma et al. [103]	Netherlands	Mixed	*ADRB3*	rs4994	1,4	2, 3, 5
Terada et al. [104]	Japan	Japanese	*RP11-382A18.1*	rs1447295 rs6983267	3,4	1, 2, 3
Thakur et al. [105]	India	Indian	*GSTT1* *GSTM1*	Null genotype Null genotype	4	1
Tigli et al. [106]	Turkey	Turkish	*CYP17*	rs743572	4	
Tsuchiya et al. [107]	Japan	Japanese	*IGF1*	CA repeat	2, 3, 4	1, 2, 4
Vijayalakshmi et al. [108]	India	South Indian	*AR*	CAG repeat GGC repeat	4	1, 2, 4
Wang et al. [109]	Japan	Japanese	*CCND1*	rs9344	2, 3	1, 2
Wang et al. [110]	Japan	Japanese	*IGFBP3*	rs2854744	3, 4	1, 2
Wang et al. [111]	Japan	Japanese	*KLK3*	rs266882 rs4802754	3, 4	1, 2
Yoo et al. [112]	Korea	Korean	*NOS2*	rs2779248 rs10459953 rs2297518	3, 4	2
Yoo et al. [113]	Korea	Korean	*IL10* *IL10RA* *IL10RB*	rs1518111 rs1554286 rs2256111 rs4252243 rs2228054 rs999788 rs2834167	3, 4	2
Zhenhua et al. [114]	Japan	Japanese	*CYP3A5*	rs776746	3, 4	1, 2, 3

BOO = bladder outlet obstruction; DRE = digital rectal examination; GWAS = genome-wide association study; PSA = prostate-specific antigen; SNP = single nucleotide polymorphism; TRUS = transrectal ultrasound.

* Assessments of descent/ethnicity/race as specified in primary publications, or from additional data from authors, or assumed for countries with low ethnic heterogeneity including Finland, Korea, and Japan.

† Listed SNPs are only those that could be included in meta-analyses. Helfand et al. [42] assessed 38 SNPs prioritized from prostate carcinoma GWAS. Gudmundsson et al. [41] assessed 15 SNPs prioritized from PSA GWAS.

‡ Same results reported for rs2234693 in Gupta et al. [64] and Sobti et al. [98].

**Fig. 2:**
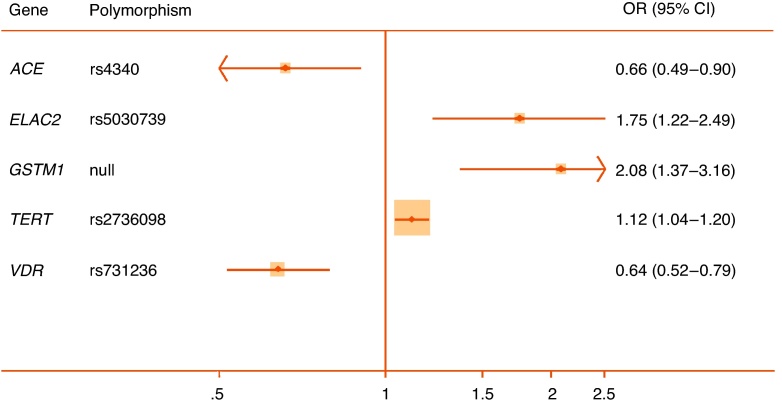
Forest plot of statistically significant single nucleotide polymorphisms in pooled analyses. Plots presented as risk associated with minor alleles. CI = confidence interval; OR = odds ratio.

Quantitative syntheses were possible for 35 polymorphisms in or near 24 genes (Table 2). Only 5 of these 35 meta-analyses achieved statistical significance (*p* < 0.05) (*ACE* rs4340, *ELAC2* rs5030739, *GSTM1* null allele, *TERT* rs2736098, and *VDR* rs731236), and of those only the rs731236 polymorphism of *VDR* could be assigned moderate epidemiological credibility (Fig. 2). The other statistically significant pooled associations were assigned weak credibility, either because of low sample sizes, high heterogeneity, or unaccounted sources of bias. In the following section we focus only on genes with at least one variant with a significant pooled association (reported in alphabetical order). Nonsignificant pooled estimates for all other genes are shown in Table 2 together with bias estimates. Corresponding forest plots are available as Supplemental Figures 1–16. A priori all nonsignificant pooled estimates were assigned weak epidemiological credibility.

**Table 2:** Interim Venice assessments of epidemiological credibility for each meta-analysis

Gene	dbSNP ID or other identifier	No. of studies	MAF sample*	Pooled OR§ (95% CI)	I^2^, %	HWE**	Proteus effect	Harbord^a^ test *p*	Risk of bias in genotyping	Risk of population stratification	Venice rating	Overall credibility
*ACE*	rs4340	2	334	0.66 (0.49–0.90)	91.9	None	Yes	NA	Low/Unknown	Low	BCA	Weak
*AR*	CAG repeat	9	4044^b^	−0.05 (−0.12 to 0.02)	47.4	NA	Yes	0.88	Low/Unknown	Possible/High‡	ACB	Weak
	GGN repeat	2	333^b^	0.12 (−0.10 to 0.34)	0.0	NA	No	NA	Low/Unknown	Possible/High‡	BCB	Weak
*CYP17*	rs743572	5	659	0.96† (0.69–1.34)	78.7	[98]	Yes	0.33	Low/Unknown	Low	ACB	Weak
*ELAC2*	rs4792311	4	675	1.02 (0.86–1.21)	1.2	[36], [100]	No	0.55	Possible/High	Low	BCC	Weak
	rs5030739	2	71	1.75 (1.22–2.49)	0.0	Unknown	No	NA	Low	Low	CBA	Weak
*FGFR2*	rs10788160	2	∼9000	1.02 (0.96–1.09)	0.0	Unknown	No	NA	Low	Low	ACA	Weak
*FGFR4*	rs351855	2	281	1.08 (0.87–1.35)	71.4	None	No	NA	Low/Unknown	Low	BCB	Weak
*GSTM1*	Null/Deletion CNV	6	695	2.08† (1.37–3.16)	74.3	NA	No	0.79	Low	Low/None	BCA	Weak
*GSTP1*	rs1695	2	192	0.93 (0.69–1.26)	0.0	None	No	No	Low/Unknown	Low	BCA	Weak
*GSTT1*	Null/deletion CNV	6	356	1.02† (0.71–1.46)	49.9	NA	Yes	0.58	Low/Unknown	Low	BCB	Weak
*HNF1B*	rs4430796	2	∼18 000	1.00 (0.93–1.07)	51.5	Unknown	No	NA	Low	Low	ACA	Weak
*IFNG*	rs2430561	2	532	0.88 (0.70–1.1)	55.2	None	No	NA	Low/Unknown	Low	BCA	Weak
*IFBP3*	rs2854744	2	731	1.14 (0.96–1.36)	27.9	None	No	NA	Low	Low	BCA	Weak
*IL10*	rs1800896	2	541	1.09 (0.86–1.38)	0.0	[71], [91]	No	NA	Possible/High	Low	BCC	Weak
*IL1RN*	rs2234663	3	510	1.64† (0.83–3.22)	87.4	Unknown	Yes	0.17	Low/Unknown	Low	BCB	Weak
*IL4*	rs2234664	2	424	0.98 (0.76–1.27)	86.6	[71], [72]	No	NA	Possible/High	Low	BCC	Weak
*KLK3*	rs266882	3	924	0.98† (0.65–1.47)	78.3	None	No	0.28	Low/Unknown	Low	BCB	Weak
	rs17632542	2	∼2000	1.08 (0.96–1.20)	0.0	Unknown	No	NA	Low	Low	ACA	Weak
	rs2735839	2	∼4000	1.06 (0.88–1.26)	0.0	Unknown	No	NA	Low	Low	ACA	Weak
*MSMB*	rs10993994	2	∼13 000	0.96 (0.91–1.02)	0.0	Unknown	No	NA	Low	Low	ACA	Weak
*RP11–38*	rs1447295	2	276	0.83 (0.60–1.14)	0.0	None	No	NA	Low/Unknown	Low	BCB	Weak
	rs6983267	2	675	1.09 (0.89–1.37)	85.5	None	Yes	NA	Low/Unknown	Low	BCB	Weak
*SRD5A2*	rs523349	4	663	1.05 (0.87–1.26)	0.0	None	No	0.21	Low/Unknown	Possible/High‡	BCC	Weak
	rs9282858	3	69	1.82† (0.83–3.98)	45.2	None	No	0.58	Low/Unknown	Possible/High‡	BCC	Weak
	TA(n) repeat	2	251	0.95 (0.73–1.23)	66.6	NA	No	NA	Unknown	Possible/High‡	BCC	Weak
*TBX3*	rs11067228	2	∼18 000	1.02 (0.97–1.08)	0.0	Unknown	No	NA	Low	Low	ACA	Weak
*TERT*	rs2736098	2	∼10 000	1.25 (1.04–1.20)	87.1	Unknown	No	NA	Low	Low	ACA	Weak
	rs401681	2	∼18 000	1.05 (0.99–1.11)	0.0	Unknown	No	NA	Low	Low	ACA	Weak
*TGFB1*	rs1800470	4	993	1.04† (0.73–1.47)	79.8	[71]	No	0.38	Low/Unknown	Low	BCB	Weak
*TNFA*	rs1800629	2	248	0.94 (0.69–1.28)	0.0	[91]	No	NA	Low/Unknown	Low	BCC	Weak
*VDR*	rs731236	5	409	0.64† (0.49–0.83)	27.2	[50]	No	0.54	Low/Unknown	Low	BBB	Moderate
	rs1544410	3	417	0.77† (0.54–1.09)	45.9	[49]	Yes	0.94	Low/Unknown	Low	BCC	Weak
	rs7975232	2	364	1.10 (0.81–1.48)	38.6	None	No	NA	Unknown	Low	BCB	Weak
	rs10735810	2	646	0.91 (0.75–1.12)	0.0	[50]	No	NA	Low	Low	BCB	Weak

HWE = Hardy-Weinberg equilibrium; MAF = minor allele frequency; OR = odds ratio; SNP = single nucleotide polymorphism.

Three-letter code corresponds to A through C ratings of the amount of evidence, its consistency, and its protection from bias (see Supplemental Fig. 1).

* Pooled sample size of participants with minor allele or nominal risk variant.

** Checked in controls and meta-analysis rechecked excluding studies with significant departure. References refer to studies with significant departure from HWE.

‡ Studies each include populations with mixed descent groups without reported adjustment.

§ SMD per copy for short tandem repeats.

a Egger test for short tandem repeats.

b Total sample size reported for short tandem repeats.

† Weights are from random effects analysis.

### 3.1 ACE

**Fig. 3:**
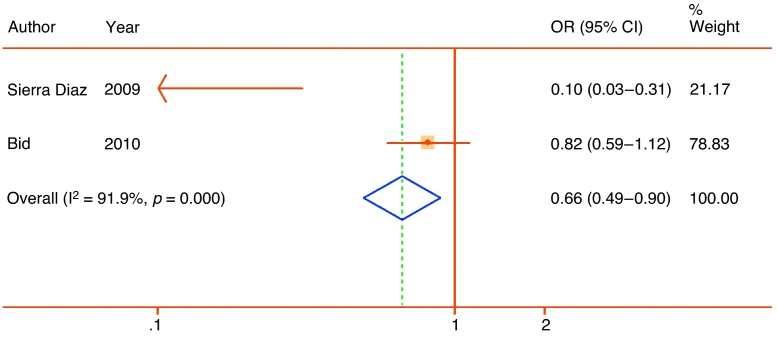
Forest plot of studies reporting associations between the rs4340 288 bp insertion polymorphism of the angiotensin-converting enzyme gene and lower urinary tract symptoms. Plot presented as risk associated with insertion allele. CI = confidence interval; OR = odds ratio.

rs4340 is an extensively studied insertion polymorphism in the angiotensin-converting enzyme gene. Although it has been suggested as a risk factor for both cardiovascular disease and a range of cancers, the most recent systematic review suggests no overall association with prostate cancer [29]. Two studies in Mexican [30] and Indian [31] populations assessed associations with LUTS or surgery for LUTS, reporting a large protective effect of the insertion (pooled OR: 0.66; 95% CI, 0.49–0.90) but with high heterogeneity (I^2^ = 91.9%) (Fig. 3). Following the recommendations of the Venice guidelines [22], the association was therefore assigned weak credibility.

### 3.2 ELAC2

**Fig. 4:**
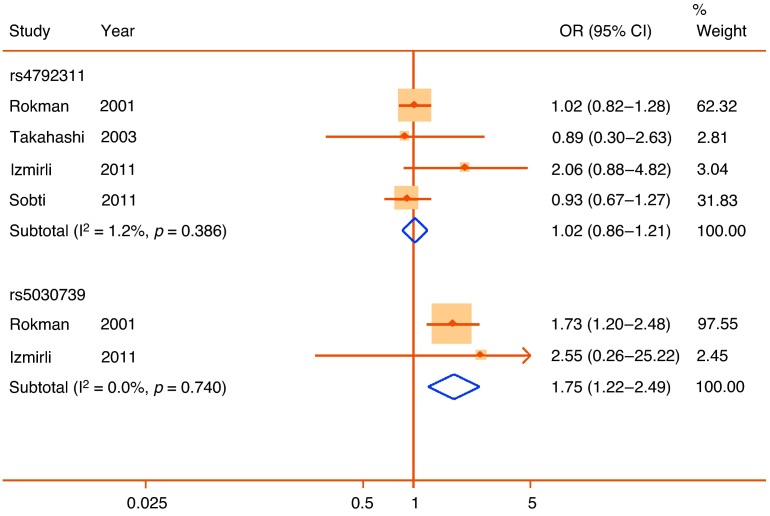
Forest plot of studies reporting associations between the rs5030739 and rs4792311 single nucleotide polymorphisms of the elaC homolog 2 (*Escherichia coli*) gene and lower urinary tract symptoms. Plot presented as risk associated with minor alleles. CI = confidence interval; OR = odds ratio.

The rs5030739 polymorphism of *ELAC2* was one of the earliest candidates as a prostate cancer risk SNP [32]. Four studies investigated an association of rs5030739 with symptomatic BPH [33], [34], [35], [36]. However, the risk SNP was found only among the two available European samples. In these Finnish and Turkish populations, the minor A allele was associated with a large increase in risk (pooled OR: 1.75; 95% CI, 1.22–2.49), with no heterogeneity (Fig. 4). There was a low risk of bias from genotyping error or population stratification. However, the meta-analysis included a low total sample of participants with the minor allele (*n* = 71), and accordingly, the association was assigned weak credibility. Analysis of a different SNP, rs4792311, in *ELAC2* in the same four studies showed nonsignificant results in all samples and no significant pooled association (Fig. 4).

### 3.3 GSTM1

**Fig. 5:**
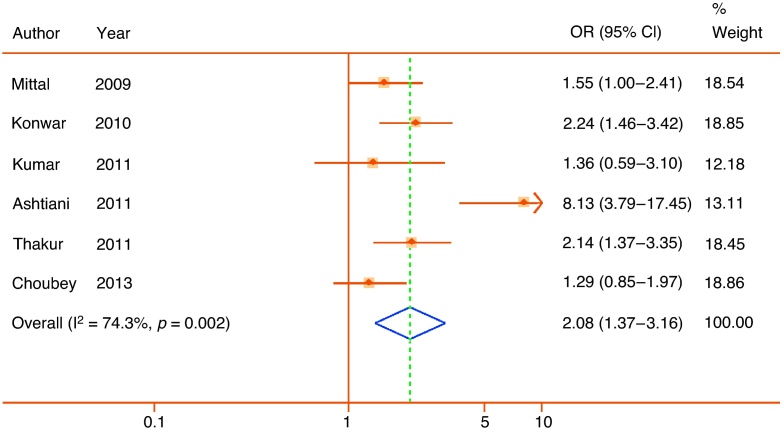
Forest plot of studies reporting associations between the null allele of the glutathione S-transferase mu 1 gene and lower urinary tract symptoms. Plot presented as risk associated with deletion/null allele. CI = confidence interval; OR = odds ratio.

The glutathione S-transferase M1 gene lies on chromosome 1 in a region with a number of common large-scale structural variants in both Asian and European populations that may delete one or both copies of the gene (null allele). The gene encodes a cytoplasmic glutathione S-transferase, involved in the detoxification of a range of compounds including potential carcinogens. Current evidence suggests the null allele is significantly associated with prostate cancer [37]. We identified the same six studies, all of Indian populations, included in a recent meta-analysis [38], with a large effect size (pooled OR: 2.08; 95% CI, 1.37–3.16) but with high heterogeneity (I^2^ = 74.3%) (Fig. 5). Although we did not identify a significant source of bias, the high heterogeneity again confers weak credibility.

### 3.4 TERT

**Fig. 6:**
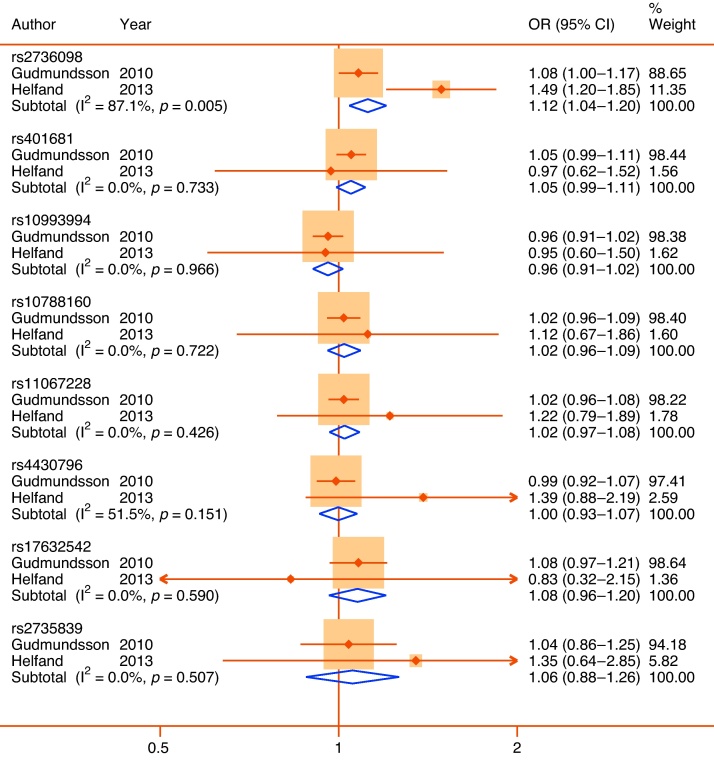
Forest plots of overlapping set of eight single nucleotide polymorphisms (SNPs) prioritized from prostate cancer or prostate-specific antigen (PSA) genome-wide association studies and tested for association with lower urinary tract symptoms (LUTS) in Icelandic (Gudmundsson et al. [41]) and US (Helfand et al. [42]) populations. Top line shows significant pooled associations between the rs2736098 SNP of the telomerase reverse transcriptase gene and LUTS. All plots presented as risk associated with minor alleles. CI = confidence interval; OR = odds ratio.

Telomerase reverse transcriptase is a catalytic subunit of telomerase that delays apoptosis. Both intronic and noncoding variants in *TERT* have been identified as prostate cancer risk SNPs in recent GWAS [39], [40]; the rs2736098 noncoding SNP has also been associated with prostate-specific antigen (PSA) variation [41]. Two large Icelandic and US studies [41], [42], including in combination >28 000 participants, tested an overlapping set of eight prostate cancer or PSA-risk SNPs prioritized from GWAS for an association with LUTS or LUTS medication use. Of these, only rs2736098 demonstrated a nominally significant pooled association but with a small effect size (OR: 1.25; 95% CI, 1.04–1.20) and high heterogeneity (I^2^ = 87.1%) (Fig. 6). Again, this confers weak epidemiological credibility.

### 3.5 VDR

**Fig. 7:**
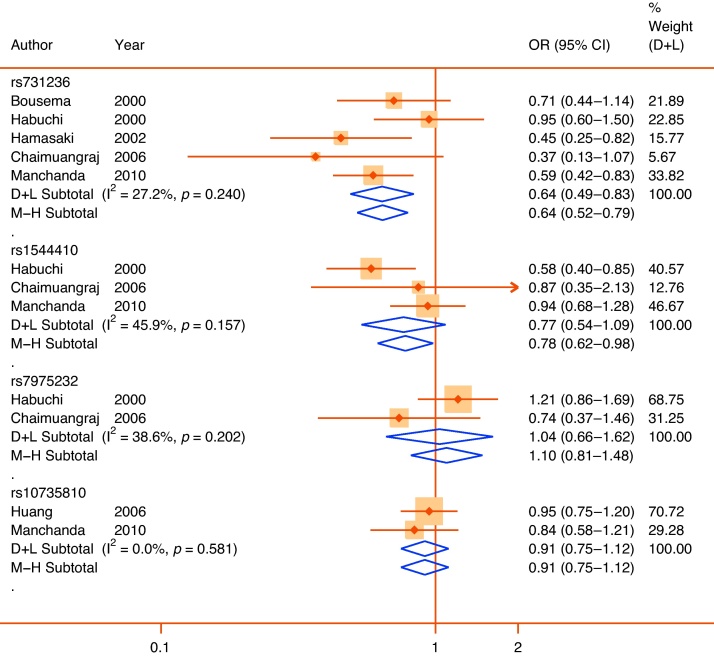
Forest plot of studies reporting associations between single nucleotide polymorphisms of the vitamin D receptor gene and lower urinary tract symptoms. Plots presented as risk associated with minor alleles. CI = confidence interval; D + L = DerSimonian & Laird; M-H = Mantel-Haenszel; OR = odds ratio.

Low vitamin D can be considered one component of the metabolic syndrome. Low vitamin D levels decrease prostate apoptosis and are associated with BPH [43]. One vitamin D3 analog has also been shown to delay prostate growth in men with BPH [44]. The vitamin D receptor has a number of common coding polymorphisms that have been investigated in association with LUTS or prostate cancer. One noncoding SNP in the 3′ region, rs731236, which is associated with increased serum vitamin D levels, may have a protective benefit against prostate cancer [45], at least in some populations. In five studies in ethnically diverse populations of its association with LUTS [46], [47], [48], [49], [50], there was a consistent protective effect of the minor allele, with a large pooled effect size (OR: 0.64; 95% CI, 0.49–0.83) and moderate heterogeneity (I^2^ = 27.2%) (Fig. 7). Uniquely among statistically significant findings, this association was both consistent across studies, with an adequate pooled sample of the minor allele (*n* = 409), and no apparent sources of bias in the primary studies, conferring moderate epidemiological credibility. One other SNP, in near perfect linkage disequilibrium, in the same gene (rs1544410) demonstrated a near significant effect in a random effects pooled analysis of Japanese, Thai, and Indian populations (OR: 0.77; 95% CI, 0.54–1.09) with moderate heterogeneity (I^2^ = 45.9%). In a prespecified analysis restricted to the two East Asian populations, the pooled effect size was large (OR: 0.62; 95% CI, 0.44–0.87), with no heterogeneity, suggesting the possibility of an effect specific to East Asian populations. Other SNPs tested in the same gene (rs7975232 and rs10735810) showed no significant pooled effects.

### 3.6 Publication and other biases

Most of the possible meta-analyses included fewer than five studies, providing low power for conventional measures of funnel plot asymmetry. Consequently, the Harbord test demonstrated no evidence of small study bias or publication bias (*p* values all >0.1) for any individual meta-analysis. However across the field as a whole, we observed a number of phenomena consistent with potential publication bias and selective reporting. The most studied polymorphism, the CAG repeat in the androgen receptor, provides a salient example. We included nine case-control studies, of which all but one contributed to meta-analysis. This meta-analysis demonstrates a marked Proteus effect [51], with the original papers based on US populations demonstrating a significant association between short CAG repeats and LUTS [52], [53], which despite repeated studies was never replicated. In this instance the initial estimates of a significant association may have resulted from unaddressed population stratification. This pattern was typical, with many studies with either obvious problems with departure from the Hardy-Weinberg equilibrium (usually with limited information about genotyping quality control), potential for population stratification, or selective use of analyses inconsistent with expected modes of inheritance (Table 2).

### 3.7 Strengths

The strengths of this review include a contemporary and comprehensive search of both published and unpublished studies, applying explicit criteria to potentially eligible studies and using standardized piloted data forms for data collection guided by written instructions, and an unbiased assessment and synthesis of reported associations. We followed a prespecified data analysis plan and successfully contacted many authors for clarifications and additional data.

### 3.8 Limitations

Among the challenges faced in this review was the inclusion of studies with varying diagnostic criteria. There is a huge disparity between symptomatic and objective findings for LUTS that is further compounded by a disparity in the standardization of terminology and diagnostic criteria used in studies. We excluded studies using only surrogate phenotypes such as PSA, prostate volume, or histology; however, we included studies with a wide range of symptomatic case definitions including definitions using extensively validated questionnaires, definitions based only on clinical interview, and definitions based on patterns of treatment seeking or use of LUTS surgery. Both LUTS in aggregate and the individual symptoms themselves may have multiple underlying causes, and these syntheses may therefore include participants not only with diverse presentations but also with diverse underlying etiologies for those symptoms.

With the exception of the studies reported in Figure 6, the meta-analyses each include <1000 participants in total and therefore provide adequate power only for associations with large effect size (approximately OR ≤0.6 or OR≥1.8). Furthermore, most meta-analyses include fewer than five studies, providing limited scope for subgroup analyses. It is therefore possible that smaller effect sizes or ethnicity-specific associations have been missed in these syntheses.

### 3.9 Implications for clinical practice

With substantial risk of bias for most replicable associations, and without clear evidence of effect modification from known environmental risks for male LUTS, it would be inadvisable to risk stratify patients on the basis of these genotypes. Neither routine nor selective use of these tests in clinical practice can currently be recommended, pending further trials.

The widespread availability of direct-to-consumer testing means some patients may present with questions about the implications of these polymorphisms. Clinicians should be not only aware of the putative risks associated with these variants, but also about the substantial uncertainty regarding these associations due to risks of bias in the primary studies.

The complexity of the pathophysiologic and pharmacologic mechanisms underlying the development of male LUTS makes them a promising target for stratified medicine. LUTS can display remarkable fluctuation over time [54], and therefore the optimal timing of intervention can be difficult to ascertain. Genetic variants can potentially provide stable and unconfounded estimates of risk of incidence or progression of LUTS. In the future, genetic counseling may therefore play one part of an investigation when considering the implications of intervention for male LUTS, and it may help target younger men for primary or secondary prevention. At this time clinicians should continue to use a family history of LUTS as a simple but powerful marker of future risk.

### 3.10 Implications for future research

The potential biases and failed replications among candidate gene studies we identify here are hardly unique to the urologic literature [55], but clear guidance now exists for the reporting and synthesis of GWAS [56]. Future studies in this field should try to minimize such catastrophic sources of bias, and urologic journals could adopt relevant reporting guidance.

Principal among the sources of imprecision identified here are inadequate sample sizes, lack of genotyping quality control, and inadequate adjustment for populations from heterogeneous descent groups. Each of these concerns could be overcome using large-scale GWAS with appropriate attention to population stratification. GWAS have been successfully used to identify many novel susceptibility variants for prostate cancer, and this technique should now be applied to male LUTS, using population-based cohorts with relevant phenotypes. The variants identified here should be prioritized for replication in future GWAS [57]. As new susceptibility variants are discovered, inclusion of DNA collections in current interventional trials in LUTS may provide significant additional power as a potential confounder.

## 4 Conclusions

Family studies and twin studies have provided convincing evidence for a genetic predisposition to male LUTS. However, despite a large research literature, this systematic review and meta-analysis using the Venice criteria has identified very few genetic variants that have been reliably replicated across populations. We found only one, the common rs731236 variant of the vitamin D receptor, credibly associated with LUTS. The currently identified genetic associations explain only a tiny fraction of the heritability. The discovery of further risk variants should both help to explain the complex pathophysiology of these symptoms and provide a route to effective primary prevention.

***Author contributions:*** Rufus Cartwright had full access to all the data in the study and takes responsibility for the integrity of the data and the accuracy of the data analysis.  

*Study concept and design:* Mangera, Cartwright, Tikkinen.

*Acquisition of data:* Mangera, Cartwright, Kirby, Tikkinen, Thiagamoorthy, Pesonen, Ambrose.

*Analysis and interpretation of data:* Cartwright, Mangera, Kirby, Tikkinen.

*Drafting of the manuscript:* Cartwright, Mangera, Kirby, Tikkinen.

*Critical revision of the manuscript for important intellectual content:* Walley, Palmer, Bennett, Khullar, Chapple, Järvelin, Rajan, Cartwright, Mangera, Kirby, Tikkinen.

*Statistical analysis:* Cartwright, Palmer, Tikkinen, Gonzalez-Maffe.

*Obtaining funding:* Cartwright, Tikkinen, Khullar, Walley, Bennett.

*Administrative, technical, or material support:* None.

*Supervision:* Walley, Palmer, Bennett, Khullar, Chapple, Järvelin.

*Other:* None.  

***Financial disclosures:*** Rufus Cartwright certifies that all conflicts of interest, including specific financial interests and relationships and affiliations relevant to the subject matter or materials discussed in the manuscript (eg, employment/affiliation, grants or funding, consultancies, honoraria, stock ownership or options, expert testimony, royalties, or patents filed, received, or pending), are the following: Chris Chapple is a speaker, consultant, and paid investigator for Astellas Pharma, Allergan, Recordati, and Pfizer. Vik Khullar is a speaker, consultant, and paid investigator for Astellas Pharma, Allergan, and Pfizer. Jori Pesonen has received unrestricted grant funding and/or travel expenses from Pfizer, Novartis, and Ferring Pharma.  

***Funding/Support and role of the sponsor:*** The study was supported by grants from the International Continence Society and the UK Medical Research Council, but neither body organization any involvement in analysis or preparation of the manuscript. The work of Kari A.O. Tikkinen was supported by unrestricted grants from the Finnish Cultural Foundation and the Finnish Medical Foundation. The work of Marjo-Riitta Järvelin was supported by an unrestricted grant from the Academy of Finland. The work of Phillip Bennett and Vik Khullar is supported by the National Institute for Health Research (NIHR) Biomedical Research Centre based at Imperial College Healthcare NHS Trust and Imperial College London.  

***Acknowledgment statement:*** The authors would like to thank the following people who provided additional information about primary studies: Oluyemi Akinloye, Yoshitaka Aoki, Basu Banerjee, Vanderlei Biolchi, Goran Brajuskovic, William Catalona, Ivana da Cruz, Nejat Dalay, Edward Giovannucci, Sezgin Gunes, Fouad Habib, Tomonori Habuchi, Jaakko Kaprio, Bart Kiemeney, Kevin McVary, Rama Mittal, Brasil Silva Neto, Leonardo Oliveira Reis, Katsushi Shibata, Manuela Simoni, Ranbir Sobti, and Teuvo Tammela.

## References

[1] Abrams P., Cardozo L., Fall M. (2002). The standardisation of terminology of lower urinary tract function: report from the Standardisation Sub-committee of the International Continence Society. Neurourol Urodyn.

[2] Sexton C.C., Coyne K.S., Kopp Z.S. (2009). The overlap of storage, voiding and postmicturition symptoms and implications for treatment seeking in the USA, UK and Sweden: EpiLUTS. BJU Int.

[3] Verhamme K.M.C., Dieleman J.P., Bleumink G.S. (2002). Incidence and prevalence of lower urinary tract symptoms suggestive of benign prostatic hyperplasia in primary care – the Triumph project. Eur Urol.

[4] Vaughan C.P., Auvinen A., Cartwright R. (2013). Impact of obesity on urinary storage symptoms: results from the FINNO Study. J Urol.

[5] Parsons J.K., Messer K., White M. (2011). Obesity increases and physical activity decreases lower urinary tract symptom risk in older men: the Osteoporotic Fractures in Men study. Eur Urol.

[6] Chapple C.R., Roehrborn C.G. (2006). A shifted paradigm for the further understanding, evaluation, and treatment of lower urinary tract symptoms in men: focus on the bladder. Eur Urol.

[7] Soler R., Andersson K.-E., Chancellor M.B. (2013). Future direction in pharmacotherapy for non-neurogenic male lower urinary tract symptoms. Eur Urol.

[8] Sanda M.G., Beaty T.H., Stutzman R.E., Childs B., Walsh P.C. (1994). Genetic susceptibility of benign prostatic hyperplasia. J Urol.

[9] Roberts R.O., Rhodes T., Panser L.A. (1995). Association between family history of benign prostatic hyperplasia and urinary symptoms: results of a population-based study. Am J Epidemiol.

[10] Kok E.T., Schouten B.W., Bohnen A.M., Groeneveld F.P., Thomas S., Bosch J.L. (2009). Risk factors for lower urinary tract symptoms suggestive of benign prostatic hyperplasia in a community based population of healthy aging men: the Krimpen Study. J Urol.

[11] Pearson J.D., Lei H.-H., Beaty T.H. (2003). Familial aggregation of bothersome benign prostatic hyperplasia symptoms. Urology.

[12] Partin A.W., Page W.F., Lee B.R., Sanda M.G., Miller R.N., Walsh P.C. (1994). Concordance rates for benign prostatic disease among twins suggest hereditary influence. Urology.

[13] Meikle A.W., Bansal A., Murray D.K., Stephenson R.A., Middleton R.G. (1999). Heritability of the symptoms of benign prostatic hyperplasia and the roles of age and zonal prostate volumes in twins. Urology.

[14] Rohrmann S., Fallin M.D., Page W.F. (2006). Concordance rates and modifiable risk factors for lower urinary tract symptoms in twins. Epidemiology.

[15] Lichtenstein P., Holm N.V., Verkasalo P.K. (2000). Environmental and heritable factors in the causation of cancer—analyses of cohorts of twins from Sweden, Denmark, and Finland. N Engl J Med.

[16] Mucci LA, Kaprio J, Harris J, et al. Heritability and familial risk of cancer: an update from the Nordic Twin Registry of Cancer (NorTwinCan). Paper presented at: American Society of Human Genetics meeting; October 22–26, 2013, Boston, MA, USA. http://ashg.org/2013meeting/abstracts/fulltext/f130123547.htm.

[17] Schenk J.M.J., Kristal A.R.A., Arnold K.B.K. (2011). Association of symptomatic benign prostatic hyperplasia and prostate cancer: results from the prostate cancer prevention trial. Am J Epidemiol.

[18] Weight C.J., Kim S.P., Jacobson D.J. (2013). The effect of benign lower urinary tract symptoms on subsequent prostate cancer testing and diagnosis. Eur Urol.

[19] Orsted D.D., Bojesen S.E., Nielsen S.F., Nordestgaard B.G. (2011). Association of clinical benign prostate hyperplasia with prostate cancer incidence and mortality revisited: a nationwide cohort study of 3,009,258 men. Eur Urol.

[20] Chokkalingam A.P.A., Nyrén O.O., Johansson J-EJ (2003). Prostate carcinoma risk subsequent to diagnosis of benign prostatic hyperplasia: a population-based cohort study in Sweden. Cancer.

[21] Parsons J.K., Schenk J.M., Arnold K.B. (2012). Finasteride reduces the risk of incident clinical benign prostatic hyperplasia. Eur Urol.

[22] Ioannidis J.P., Boffetta P., Little J. (2007). Assessment of cumulative evidence on genetic associations: interim guidelines. Int J Epidemiol.

[23] Harbord R.M., Egger M., Sterne J.A.C. (2006). A modified test for small-study effects in meta-analyses of controlled trials with binary endpoints. Stat Med.

[24] Egger M., Davey-Smith G., Schneider M., Minder C. (1997). Bias in meta-analysis detected by a simple, graphical test. BMJ.

[25] Little J, Higgins J, editors. The HuGENet™ HuGE review handbook, v.1.0; 2006. http://www.med.uottawa.ca/public-health-genomics/web/assets/documents/HuGE_Review_Handbook_V1_0.pdf.

[26] Moher D., Liberati A., Tetzlaff J., Altman D.G., PRISMA Group (2009). Preferred reporting items for systematic reviews and meta-analyses: the PRISMA statement. Ann Intern Med.

[27] Takeda M, Araki I, Kamiyama M, Takihana Y, Tanabe N. Single nucleotide polymorphism of alpha1a and beta3-adrenoceptors in urological patients with and without micturition symptoms—possible mechanism for hyperactivity of adrenergic nerve and tailor-made medicine. Paper presented at: 32nd Annual Meeting of the International Continence Society; August 2002; Heidelberg, Germany.

[28] Schwanke C.H.A., Bittencourt L., Noronha J.A.P., Augustin S.A.J., Jung I.E., Cruz I.B.M. (2007). Is there an association between T102C polymorphism of the serotonin receptor 2A gene and urinary incontinence?. Braz J Med Biol Res.

[29] Zhang Y.Y., He J.J., Deng Y.Y. (2011). The insertion/deletion (I/D) polymorphism in the angiotensin-converting enzyme gene and cancer risk: a meta-analysis. BMC Med Genet.

[30] Sierra Diaz E., Sanchez Corona J., Rosales Gómez R.C. (2009). Angiotensin-converting enzyme insertion/deletion and angiotensin type 1 receptor A1166C polymorphisms as genetic risk factors in benign prostatic hyperplasia and prostate cancer. J Renin Angiotensin Aldosterone Syst.

[31] Bid H.K., Manchanda P.K., Konwar R., Hanif K., Nayak V.L., Singh V. (2010). Does angiotensin-converting enzyme polymorphism have association with symptomatic benign prostatic hyperplasia?. Indian J Urol.

[32] Xu B., Tong N., Li J.M., Zhang Z.D., Wu H.F. (2010). ELAC2 polymorphisms and prostate cancer risk: a meta-analysis based on 18 case-control studies. Prostate Cancer Prostatic Dis.

[33] Rökman A.A., Ikonen T.T., Mononen N.N. (2001). ELAC2/HPC2 involvement in hereditary and sporadic prostate cancer. Cancer Res.

[34] Takahashi H.H., Lu W., Watanabe M. (2003). Ser217Leu polymorphism of the HPC2/ELAC2 gene associated with prostatic cancer risk in Japanese men. Int J Cancer.

[35] Izmirli M., Arikan B., Bayazit Y., Alptekin D. (2011). Associations of polymorphisms in HPC2/ELAC2 and SRD5A2 genes with benign prostate hyperplasia in Turkish men. Asian Pac J Cancer Prev.

[36] Sobti R.C., Thakur H., Gupta L., Janmeja A.K., Seth A., Singh S.K. (2011). Polymorphisms in the HPC/ELAC-2 and alpha 1-antitrypsin genes that correlate with human diseases in a North Indian population. Mol Biol Rep.

[37] Mo Z., Gao Y., Cao Y., Gao F., Jian L. (2009). An updating meta-analysis of the GSTM1, GSTT1, and GSTP1 polymorphisms and prostate cancer: a HuGE review. Prostate.

[38] Choubey V.K., Sankhwar S.N., Tewari R., Sankhwar P., Singh B.P., Rajender S. (2012). Null genotypes at the GSTM1and GSTT1genes and the risk of benign prostatic hyperplasia: a case-control study and a meta-analysis. Prostate.

[39] Kote-Jarai Z., Olama A.A., Giles G. (2011). Seven prostate cancer susceptibility loci identified by a multi-stage genome-wide association study. Nat Genet.

[40] Rafnar T., Sulem P., Stacey S.N. (2009). Sequence variants at the TERT-CLPTM1L locus associate with many cancer types. Nat Genet.

[41] Gudmundsson J., Besenbacher S., Sulem P. (2010). Genetic correction of PSA values using sequence variants associated with PSA levels. Sci Transl Med.

[42] Helfand B.T., Hu Q., Loeb S., McVary K.T., Catalona W.J. (2013). Genetic sequence variants are associated with severity of lower urinary tract symptoms and prostate cancer susceptibility. J Urol.

[43] Haghsheno M.A., Mellström D., Behre C.J. (2013). Low 25-OH vitamin D is associated with benign prostatic hyperplasia. J Urol.

[44] Colli E., Rigatti P., Montorsi F. (2006). BXL628, a novel vitamin D3 analog, arrests prostate growth in patients with benign prostatic hyperplasia: a randomized clinical trial. Eur Urol.

[45] Guo Y.J., Shi Z.M., Liu J.D., Lei N., Chen Q.H., Tang Y. (2012). Meta-analysis of the relation between the VDR gene TaqI polymorphism and genetic susceptibility to prostate cancer in Asian populations. Asian Pac J Cancer Prev.

[46] Bousema J.T., Bussemakers M.J., van Houwelingen K.P. (2000). Polymorphisms in the vitamin D receptor gene and the androgen receptor gene and the risk of benign prostatic hyperplasia. Eur Urol.

[47] Habuchi T., Suzuki T., Sasaki R. (2000). Association of vitamin D receptor gene polymorphism with prostate cancer and benign prostatic hyperplasia in a Japanese population. Cancer Res.

[48] Hamasaki T., Inatomi H., Katoh T., Ikuyama T., Matsumoto T. (2002). Significance of vitamin D receptor gene polymorphism for risk and disease severity of prostate cancer and benign prostatic hyperplasia in Japanese. Urol Int.

[49] Chaimuangraj S., Thammachoti R., Ongphiphadhanakul B., Thammavit W. (2006). Lack of association of VDR polymorphisms with Thai prostate cancer as compared with benign prostate hyperplasia and controls. Asian Pac J Cancer Prev.

[50] Manchanda P.K., Konwar R., Nayak V.L., Singh V., Bid H.K. (2010). Association of genetic variants of the vitamin D receptor (VDR) gene (Fok-I, Taq-I and Bsm-I) with susceptibility of benign prostatic hyperplasia in a North Indian population. Asian Pac J Cancer Prev.

[51] Ioannidis J.P., Trikalinos T.A. (2005). Early extreme contradictory estimates may appear in published research: the Proteus phenomenon in molecular genetics research and randomized trials. J Clin Epidemiol.

[52] Giovannucci E., Platz E.A., Stampfer M.J. (1999). The CAG repeat within the androgen receptor gene and benign prostatic hyperplasia. Urology.

[53] Giovannucci E., Stampfer M.J., Chan A. (1999). CAG repeat within the androgen receptor gene and incidence of surgery for benign prostatic hyperplasia in U.S. physicians. Prostate.

[54] Vaughan C.P., Johnson T.M., Haukka J. (2014). The fluctuation of nocturia among men with lower urinary tract symptoms allocated to placebo during a 12 month randomized controlled trial. J Urol.

[55] Colhoun H.M., McKeigue P.M., Davey-Smith G. (2003). Problems of reporting genetic associations with complex outcomes. Lancet.

[56] Little J., Higgins J.P.T., Ioannidis J.P.A. (2009). STrengthening the REporting of Genetic Association Studies (STREGA)—an extension of the STROBE statement. Genet Epidemiol.

[57] Siontis K.C.M., Patsopoulos N.A., Ioannidis J.P.A. (2010). Replication of past candidate loci for common diseases and phenotypes in 100 genome-wide association studies. Eur J Hum Genet.

[58] Ashtiani Z.O., Hasheminasab S.M., Ayati M., Goulian B.S., Modarressi M.H. (2011). Are GSTM1, GSTT1 and CAG repeat length of androgen receptor gene polymorphisms associated with risk of prostate cancer in Iranian patients?. Pathol Oncol Res.

[59] Berhane N., Sobti R.C., Mahdi S.A. (2012). DNA repair genes polymorphism (XPG and XRCC1) and association of prostate cancer in a north Indian population. Mol Biol Rep.

[60] Biolchi V., Silva Neto B., Koff W., Brum I.S. (2012). Androgen receptor CAG polymorphism and the risk of benign prostatic hyperplasia in a Brazilian population. Int Braz J Urol.

[61] Biolchi V., Silva Neto B., Pianta D.B., Koff W.J., Berger M., Brum I.S. (2012). Androgen receptor GGC polymorphism and testosterone levels associated with high risk of prostate cancer and benign prostatic hyperplasia. Mol Biol Rep.

[62] Faria P.C., Saba K., Neves A.F. (2007). Transforming growth factor-beta 1 gene polymorphisms and expression in the blood of prostate cancer patients. Cancer Invest.

[63] Gunes S., Bagci H., Sarikaya S., Bilen C.Y., Kara N. (2007). Prostate-specific antigen and 17-hydroxylase polymorphic genotypes in patients with prostate cancer and benign prostatic hyperplasia. DNA Cell Biol.

[64] Gupta L., Thakur H., Sobti R.C., Seth A., Singh S.K. (2010). Role of genetic polymorphism of estrogen receptor-alpha gene and risk of prostate cancer in north Indian population. Mol Cell Biochem.

[65] Habuchi T., Liqing Z., Suzuki T. (2000). Increased risk of prostate cancer and benign prostatic hyperplasia associated with a CYP17 gene polymorphism with a gene dosage effect. Cancer Res.

[66] Ho C.K., Anwar S., Nanda J., Habib F.K. (2010). FGFR4 Gly388Arg polymorphism and prostate cancer risk in Scottish men. Prostate Cancer Prostatic Dis.

[67] Huang S.P., Huang C.Y., Wu W.J. (2006). Association of vitamin D receptor FokI polymorphism with prostate cancer risk, clinicopathological features and recurrence of prostate specific antigen after radical prostatectomy. Int J Cancer.

[68] Huang S.P., Huang C.H., Wu W.J. (2004). p53 Codon 72 and p21 codon 31 polymorphisms in prostate cancer. Cancer Epidemiol Biomarkers Prev.

[69] Jerónimo C., Varzim G., Henrique R. (2002). I105 V polymorphism and promoter methylation of the GSTP1 gene in prostate adenocarcinoma. Cancer Epidemiol Biomarkers Prev.

[70] Kamoto T., Isogawa Y., Shimizu Y. (2005). Association of a genetic polymorphism of the E-cadherin gene with prostate cancer in a Japanese population. Jpn J Clin Oncol.

[71] Kesarwani P., Mittal R.D. (2010). Association of pro/anti-inflammatory cytokine gene polymorphisms with benign prostate hyperplasia risk. Indian J Clin Biochem.

[72] Konwar R., Gara R., Singh M., Singh V., Chattopadhyay N., Bid H.K. (2008). Association of interleukin-4 and interleukin-1 receptor antagonist gene polymorphisms and risk of benign prostatic hyperplasia. Urology.

[73] Konwar R., Manchanda P.K., Chaudhary P., Nayak V.L., Singh V., Bid H.K. (2010). Glutathione S-transferase gene variants and risk of benign prostate hyperplasia in a North Indian population. Asian Pac J Cancer Prev.

[74] Kristal A.R., Price D.K., Till C. (2010). Androgen receptor CAG repeat length is not associated with the risk of incident symptomatic benign prostatic hyperplasia: results from the Prostate Cancer Prevention Trial. Prostate.

[75] Kumar V., Yadav C.S., Datta S.K. (2011). Association of GSTM1 and GSTT1 polymorphism with lipid peroxidation in benign prostate hyperplasia and prostate cancer: a pilot study. Dis Markers.

[76] Kumazawa T., Tsuchiya N., Wang L. (2004). Microsatellite polymorphism of steroid hormone synthesis gene CYP11A1 is associated with advanced prostate cancer. Int J Cancer.

[77] Li C., Grönberg H., Matsuyama H. (2003). Difference between Swedish and Japanese men in the association between AR CAG repeats and prostate cancer suggesting a susceptibility-modifying locus overlapping the androgen receptor gene. Int J Mol Med.

[78] Li Z., Habuchi T., Mitsumori K. (2003). Association of V89L SRD5A2 polymorphism with prostate cancer development in a Japanese population. J Urol.

[79] Li Z., Habuchi T., Tsuchiya N. (2004). Increased risk of prostate cancer and benign prostatic hyperplasia associated with transforming growth factor-beta 1 gene polymorphism at codon10. Carcinogenesis.

[80] Licastro F., Bertaccini A., Porcellini E. (2008). Alpha 1 antichymotrypsin genotype is associated with increased risk of prostate carcinoma and PSA levels. Anticancer Res.

[81] Ma Z., Tsuchiva N., Yuasa T. (2008). Polymorphisms of fibroblast growth factor receptor 4 have association with the development of prostate cancer and benign prostatic hyperplasia and the progression of prostate cancer in a Japanese population. Int J Cancer.

[82] Madigan M.P., Gao Y.T., Deng J. (2003). CYP17 polymorphisms in relation to risks of prostate cancer and benign prostatic hyperplasia: a population-based study in China. Int J Cancer.

[83] Mitsumori K., Terai A., Oka H. (1999). Androgen receptor CAG repeat length polymorphism in benign prostatic hyperplasia (BPH): correlation with adenoma growth. Prostate.

[84] Mittal R.D., Mishra D.K., Bid H.K., Mandhani A. (2004). Interleukin-1 receptor antagonist polymorphism in patients with prostate cancer and benign prostatic hyperplasia: a case control study from north India. UroOncology.

[85] Mittal R.D., Kesarwani P., Singh R., Ahirwar D., Mandhani A. (2009). GSTM1, GSTM3 and GSTT1 gene variants and risk of benign prostate hyperplasia in North India. Dis Markers.

[86] Mononen N., Ikonen T., Autio V. (2002). Androgen receptor CAG polymorphism and prostate cancer risk. Hum Genet.

[87] Mononen N., Ikonen T., Syrjäkoski K. (2001). A missense substitution A49T in the steroid 5-alpha-reductase gene (SRD5A2) is not associated with prostate cancer in Finland. Br J Cancer.

[88] Narita S., Tsuchiva N., Wang L. (2004). Association of lipoprotein lipase gene polymorphism with risk of prostate cancer in a Japanese population. Int J Cancer.

[89] Nikolić Z.Z., Brajušković G.N., Pavićević D.L.J. (2013). Assessment of possible association between rs3787016 and prostate cancer risk in Serbian population. Int J Clin Exp Med.

[90] Omrani M.D., Taghipour-Bazargani S., Salari-Lak S., Bagheri M. (2009). Association of codon 10 polymorphism of the transforming growth factor beta 1 gene with prostate cancer and hyperplasia in an Iranian population. Urol Int.

[91] Omrani M.D., Bazargani S., Bagheri M. (2008). Interlukin-10, interferon-γ and tumor necrosis factor-α genes variation in prostate cancer and benign prostatic hyperplasia. Curr Urol.

[92] Rajender S., Vijayalakshmi K., Pooja S. (2009). Longer (TA)n repeat but not A49T and V89L polymorphisms in SRD5A2 gene may confer prostate cancer risk in South Indian men. J Androl.

[93] Rökman A., Ikonen T., Seppälä E.H. (2002). Germline alterations of the RNASEL gene, a candidate HPC1 gene at 1q25, in patients and families with prostate cancer. Am J Hum Genet.

[94] Safarinejad M.R., Shafiei N., Safarinejad S. (2011). Relationship of insulin-like growth factor (IGF) binding protein-3 (IGFBP-3) gene polymorphism with the susceptibility to development of prostate cancer and influence on serum levels of IGF-I, and IGFBP-3. Growth Horm IGF Res.

[95] Salam M.T., Ursin G., Skinner E.C., Dessissa T., Reichardt J.K. (2005). Associations between polymorphisms in the steroid 5-alpha reductase type II (SRD5A2) gene and benign prostatic hyperplasia and prostate cancer. Urol Oncol.

[96] Seppälä E.H., Autio V., Duggal P. (2007). *KLF6* IVS1-27G>A variant and the risk of prostate cancer in Finland. Eur Urol.

[97] Shibata K., Hirasawa A., Moriyama N., Kawabe K., Ogawa S., Tsujimoto G. (1996). Alpha 1a-adrenoceptor polymorphism: pharmacological characterization and association with benign prostatic hypertrophy. Br J Pharmacol.

[98] Sobti R.C., Gupta L., Singh S.K. (2008). Role of hormonal genes and risk of prostate cancer: gene-gene interactions in a North Indian population. Cancer Genet Cytogenet.

[99] Steiner M., Hillenbrand M., Borkowsi M., Seiter H., Schuff-Werner P. (1999). 609 C --> T polymorphism in NAD(P)H:quinone oxidoreductase gene in patients with prostatic adenocarcinoma or benign prostatic hyperplasia. Cancer Lett.

[100] Takahashi H., Lu W., Watanabe M. (2003). Ser217Leu polymorphism of the HPC2/ELAC2 gene associated with prostatic cancer risk in Japanese men. Int J Cancer.

[101] Tanaka Y., Sasaki M., Shiina H. (2006). Catechol-O-methyltransferase gene polymorphisms in benign prostatic hyperplasia and sporadic prostate cancer. Cancer Epidemiol Biomarkers Prev.

[102] Tanaka Y., Zaman N.S., Majid S. (2009). Polymorphisms of MLH1 in benign prostatic hyperplasia and sporadic prostate cancer. Biochem Biophys Res Commun.

[103] Teitsma C.A., de la Rosette de J.J., Michel M.C. (2012). Are polymorphisms of the β 3-adrenoceptor gene associated with an altered bladder function?. Neurourol Urodyn.

[104] Terada N., Tuschiya N., Ma Z. (2008). Association of genetic polymorphisms at 8q24 with the risk of prostate cancer in a Japanese population. Prostate.

[105] Thakur H., Gupta L., Sobti R.C., Janmeja A.K., Seth A., Singh S.K. (2011). Association of GSTM1T1 genes with COPD and prostate cancer in north Indian population. Mol Biol Rep.

[106] Tigli H., Yazici H., Dalay N. (2003). Cyp17 genetic polymorphism in prostate cancer and benign prostatic hyperplasia. Res Commun Mol Pathol Pharmacol.

[107] Tsuchiya N., Wang L., Horikawa Y. (2005). CA repeat polymorphism in the insulin-like growth factor-I gene is associated with increased risk of prostate cancer and benign prostatic hyperplasia. Int J Oncol.

[108] Vijayalakshmi K., Thangaraj K., Rajender S. (2006). GGN repeat length and GGN/CAG haplotype variations in the androgen receptor gene and prostate cancer risk in South Indian men. J Hum Genet.

[109] Wang L., Habuchi T., Mitsumori K. (2003). Increased risk of prostate cancer associated with AA genotype of cyclin D1 gene A870G polymorphism. Int J Cancer.

[110] Wang L., Habuchi T., Tsuchiya N. (2003). Insulin-like growth factor-binding protein-3 gene -202 A/C polymorphism is correlated with advanced disease status in prostate cancer. Cancer Res.

[111] Wang L.Z., Sato K., Tsuchiya N. (2003). Polymorphisms in prostate-specific antigen (PSA) gene, risk of prostate cancer, and serum PSA levels in Japanese population. Cancer Lett.

[112] Yoo K.H., Kim S.K., Chung J.H., Chang S.G. (2010). Nitric oxide synthase 2 gene polymorphisms are associated with prostatic volume in Korean men with benign prostatic hyperplasia. Asian J Androl.

[113] Yoo K.H., Kim S.K., Chung J.H., Chang S.G. (2011). Association of IL10, IL10RA, and IL10RB polymorphisms with benign prostate hyperplasia in Korean population. J Korean Med Sci.

[114] Zhenhua L., Tsuchiya N., Narita S. (2005). CYP3A5 gene polymorphism and risk of prostate cancer in a Japanese population. Cancer Lett.

