# Supplementary material for: Systematic Review and Meta-analysis of Candidate Gene Association Studies of Lower Urinary Tract Symptoms in Men[image]
Source: Eur Urol. 2014 Oct;66(4):752–68. doi: 10.1016/j.eururo.2014.01.007 (PMC4410299; doi:10.1016/j.eururo.2014.01.007)
Supplement: Supplementary file 1 [file mmc1.doc]

**Supplemental Table 1 – Functional annotation of investigated genes** **(functions assigned using http://david.abcc.ncifcrf.gov)**

| Official  Gene Symbol | Gene Name(s) | Gene Ontology Molecular Function(s) |
| --- | --- | --- |
| *ACE* | angiotensin I converting enzyme (peptidyl-dipeptidase A) 1 | metallopeptidase activity, exopeptidase activity, bradykinin receptor binding, transition metal ion binding |
| *ADRA1A* | adrenergic, alpha-1A-, receptor | molecular transducer activity, G-protein coupled receptor activity, |
| *ADRB3* | adrenergic, beta-3-, receptor | molecular transducer activity, G-protein coupled receptor activity, adrenergic receptor binding |
| *AGTR1* | angiotensin II receptor, type 1 | angiotensin receptor activity, peptide receptor activity |
| *AR* | androgen receptor | transcription regulator activity, molecular transducer activity, steroid hormone receptor activity, transition metal ion binding |
| *CCND1* | cyclin D1 | enzyme regulator activity, protein kinase activity, protein kinase binding |
| *CDH1* | cadherin 1, type 1, E-cadherin (epithelial) | transcription regulator activity, calcium ion binding, protein phosphatase binding |
| *CDKN1A* | cyclin-dependent kinase inhibitor 1A (p21, Cip1) | enzyme regulator activity, cyclin-dependent protein kinase inhibitor activity, protein serine/threonine kinase inhibitor activity, transition metal ion binding |
| *CLPTM1L* | cleft lip and palate transmembrane protein 1-like protein | apoptosis, programmed cell death |
| *COMT* | catechol-O-methyltransferase | magnesium ion binding, O-methyltransferase activity, S-adenosylmethionine-dependent methyltransferase activity |
| *CTBP2* | C-terminal binding protein 2 | transcription regulator activity, transcription corepressor activity, |
| *CYP11A1* | cytochrome P450, family 11, subfamily A, polypeptide 1 | electron carrier activity, cholesterol monooxygenase (side-chain-cleaving) activity, cholesterol binding, transition metal ion binding |
| *CYP17A1* | cytochrome P450, family 17, subfamily A, polypeptide 1 | steroid 17-alpha-monooxygenase activity, iron ion binding, steroid hydroxylase activity, electron carrier activity, heme binding, tetrapyrrole binding, transition metal ion binding |
| *CYP3A5* | cytochrome P450, family 3, subfamily A, polypeptide 5 | electron carrier activity, transition metal ion binding, aromatase activity |
| *DAB2IP* | DAB2 interacting protein | enzyme regulator activity, |
| *EEFSEC* | eukaryotic elongation factor, selenocysteine-tRNA-specific | pyrophosphatase activity, guanyl ribonucleotide binding, |
| *EHBP1* | EH domain binding protein 1 | binding |
| *ELAC2* | elaC homolog 2 (E. coli) | endonuclease activity, transition metal ion binding, |
| *ERCC5* | excision repair cross-complementing rodent repair deficiency, complementation group | DNA binding, single-stranded DNA binding, nuclease activity, endonuclease activity, structure-specific DNA binding |
| *ESR1* | estrogen receptor 1 | enzyme regulator activity, transcription regulator activity, molecular transducer activity, steroid hormone receptor activity, promoter binding, transition metal ion binding |
| *FGFR2* | fibroblast growth factor receptor 2 | molecular transducer activity, protein kinase activity, heparin binding, adenyl ribonucleotide binding |
| *FGFR4* | fibroblast growth factor receptor 4 | molecular transducer activity, adenyl ribonucleotide binding, protein kinase activity |
| *GSTM1* | glutathione S-transferase mu 1 | catalytic activity |
| *GSTM3* | glutathione S-transferase mu 3 (brain) | catalytic activity |
| *GSTP1* | glutathione S-transferase pi 1 | catalytic activity |
| *GSTT1* | glutathione S-transferase theta 1 | catalytic activity |
| *HNF1B* | HNF1 homeobox B | transcription regulator activity |
| *HTR2A* | 5-hydroxytryptamine (serotonin) receptor 2A | molecular transducer activity, G-protein coupled receptor activity |
| *IFNG* | interferon, gamma | interferon-gamma receptor binding |
| *IGF1* | insulin-like growth factor 1 (somatomedin C) | neuropeptide hormone activity |
| *IGFBP3* | insulin-like growth factor binding protein 3 | enzyme regulator activity, protein tyrosine phosphatase activator activity, insulin-like growth factor I binding |
| *IL10* | interleukin 10 | interleukin-10 receptor binding |
| *IL10RA* | interleukin 10 receptor, alpha | molecular transducer activity, cytokine receptor activity, interleukin-10 receptor activity |
| *IL10RB* | interleukin 10 receptor, beta | molecular transducer activity, cytokine receptor activity, interleukin-10 receptor activity, |
| *IL1B* | interleukin 1, beta | interleukin-1 receptor binding |
| *IL1RN* | interleukin 1 receptor antagonist | molecular transducer activity, interleukin-1 receptor binding, fibroblast growth factor receptor antagonist activity, receptor inhibitor activity |
| *IL4* | interleukin 4 | interleukin-4 receptor binding |
| *IL6* | interleukin 6 (interferon, beta 2) | interleukin-6 receptor binding |
| *ITGA6* | integrin, alpha 6 | molecular transducer activity, calcium ion binding, |
| *JAZF1* | JAZF zinc finger 1 | transcription regulator activity, transcription corepressor activity, transition metal ion binding, |
| *KLF6* | Kruppel-like factor 6 | transcription regulator activity, double-stranded DNA binding, transition metal ion binding, |
| *KLK3* | kallikrein-related peptidase 3 | endopeptidase activity, serine-type endopeptidase activity, serine-type peptidase activity |
| *LMTK2* | lemur tyrosine kinase 2 | enzyme regulator activity, protein kinase activity, phosphoprotein phosphatase inhibitor activity, adenyl ribonucleotide binding |
| *LPL* | lipoprotein lipase | lipoprotein lipase activity, phospholipase activity, triacylglycerol lipase activity, heparin binding, |
| *MLH1* | mutL homolog 1, colon cancer, nonpolyposis type 2 (E. coli) | double-stranded DNA binding, single-stranded DNA binding, MutSalpha complex binding, adenyl ribonucleotide binding, |
| *MSMB* | microseminoprotein, beta | no assigned GO Molecular functions |
| *NOS2* | nitric oxide synthase 2, inducible | electron carrier activity, enzyme regulator activity, nitric-oxide synthase activity, calcium ion binding, FMN binding, transition metal ion binding, FAD binding |
| *NQO1* | NAD(P)H dehydrogenase, quinone 1 | electron carrier activity, NAD(P)H dehydrogenase (quinone) activity, cytochrome-b5 reductase activity |
| *NUDT11* | nudix (nucleoside diphosphate linked moiety X)-type motif 11 | magnesium ion binding, pyrophosphatase activity, transition metal ion binding |
| *PCAT1* | prostate cancer associated transcript 1 | no assigned GO Molecular functions |
| *PDLIM5* | PDZ and LIM domain 5 | protein kinase binding, transition metal ion binding, |
| *RNASEL* | ribonuclease L (2',5'-oligoisoadenylate synthetase-dependent) | endonuclease activity, ribonuclease activity, protein kinase activity, adenyl ribonucleotide binding, transition metal ion binding |
| *SERPINA1* | serpin peptidase inhibitor, clade A (alpha-1 antiproteinase, antitrypsin), member 1 | enzyme regulator activity, serine-type endopeptidase inhibitor activity |
| *SERPINA3* | serpin peptidase inhibitor, clade A (alpha-1 antiproteinase, antitrypsin), member 3 | enzyme regulator activity, serine-type endopeptidase inhibitor activity |
| *SLC22A3* | solute carrier family 22 (extraneuronal monoamine transporter), member 3 | transporter activity, cation transmembrane transporter activity, quaternary ammonium group transmembrane transporter activity |
| *SPINT2* | serine peptidase inhibitor, Kunitz type, 2 | enzyme regulator activity, serine-type endopeptidase inhibitor activity |
| *SRD5A2* | steroid-5-alpha-reductase, alpha polypeptide 2 (3-oxo-5 alpha-steroid delta 4-dehydrogenase alpha 2) | 3-oxo-5-alpha-steroid 4-dehydrogenase activity |
| *TBX3* | T-box 3 | transcription regulator activity |
| *TERT* | telomerase reverse transcriptase | DNA polymerase activity, telomeric DNA binding |
| *TGFB1* | transforming growth factor, beta 1 | transcription regulator activity |
| *THADA* | thyroid adenoma associated | molecular transducer activity |
| *TNF* | tumor necrosis factor | cytokine activity, tumor necrosis factor receptor binding, tumor necrosis factor receptor superfamily binding |
| *TNRC6B* | trinucleotide repeat containing 6B | double-stranded DNA binding, single-stranded DNA binding, promoter binding, adenyl ribonucleotide binding, transition metal ion binding |
| *TP53* | tumor protein p53 | transcription regulator activity, steroid hormone receptor activity, vitamin D3 receptor activity, promoter binding, nuclear hormone receptor binding, transition metal ion binding, retinoid X receptor binding |
| *VDR* | vitamin D (1,25- dihydroxyvitamin D3) receptor | transcription regulator activity, molecular transducer activity |
| *XRCC1* | X-ray repair complementing defective repair in Chinese hamster cells 1 | binding |
