# Supplementary material for: Systematic Review and Meta-analysis of Candidate Gene Association Studies of Lower Urinary Tract Symptoms in Men[image]
Source: Eur Urol. 2014 Oct;66(4):752–68. doi: 10.1016/j.eururo.2014.01.007 (PMC4410299; doi:10.1016/j.eururo.2014.01.007)
Supplement: Supplementary file 3 [file mmc3.doc]

**Supplemental Fig. 1 – Forest plot of studies* reporting associations between the CAG repeat polymorphism of the androgen receptor gene and lower urinary tract symptoms.**

* Only studies reporting risk per repeat copy included, and plot presented as risk per copy.


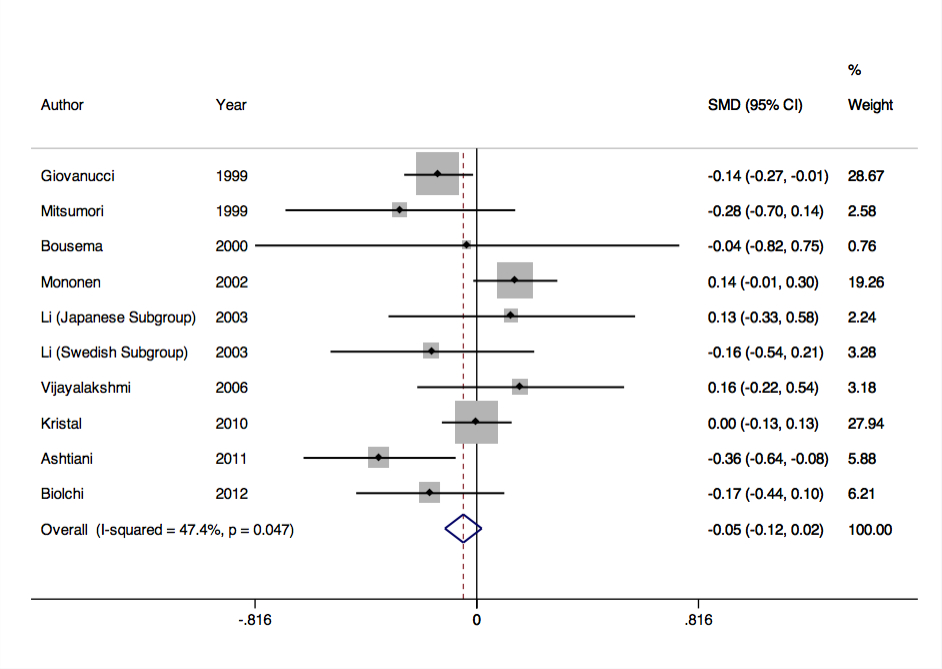


**Supplemental Fig. 2 – Forest plot of studies* reporting associations between the GGN repeat polymorphism of the androgen receptor gene and lower urinary tract symptoms.**

* Only studies reporting risk per repeat copy included, and plot presented as risk per copy.


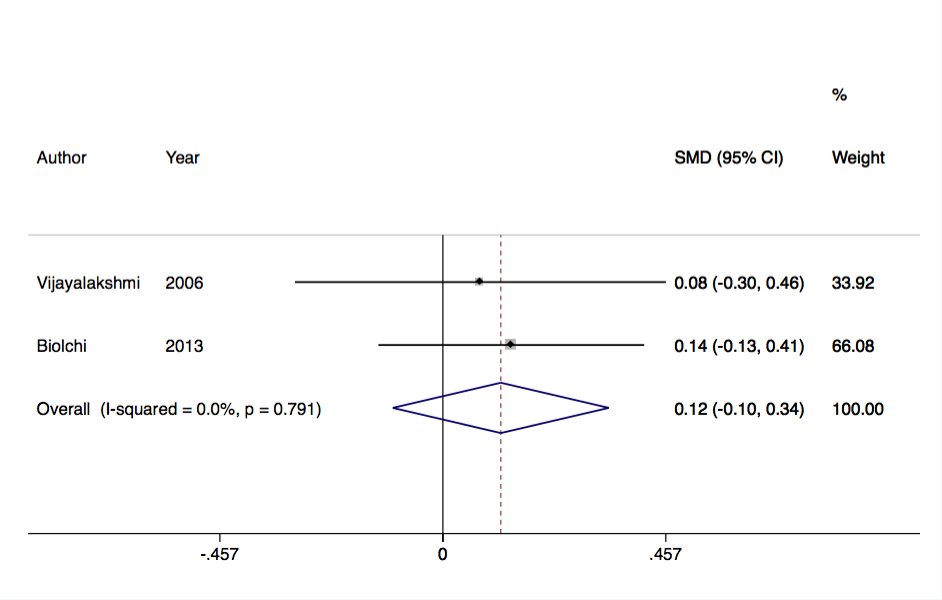


**Supplemental Fig. 3 – Forest plot of studies reporting associations between the rs743572 single nucleotide polymorphism (SNP) of the CYP17 gene and lower urinary tract symptoms. RefSNP alleles A/G. Plot presented as risk associated with minor A allele.**


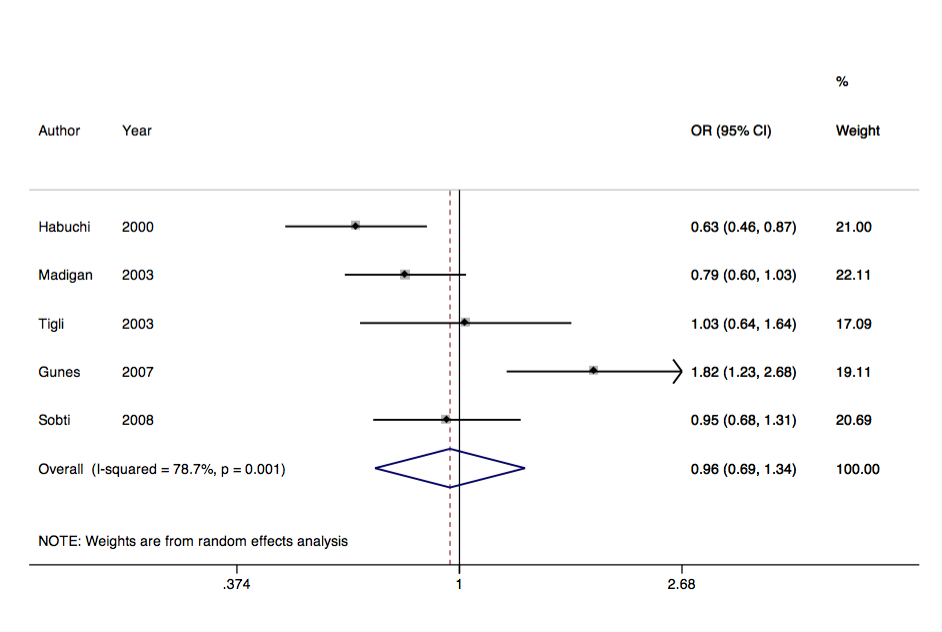


**Supplemental Fig. 4 – Forest plot of studies reporting associations between the rs351855 single nucleotide polymorphism (SNP) of the fibroblast growth factor receptor 4 gene and lower urinary tract symptoms. RefSNP alleles C/T. Plot presented as risk associated with minor T allele.**


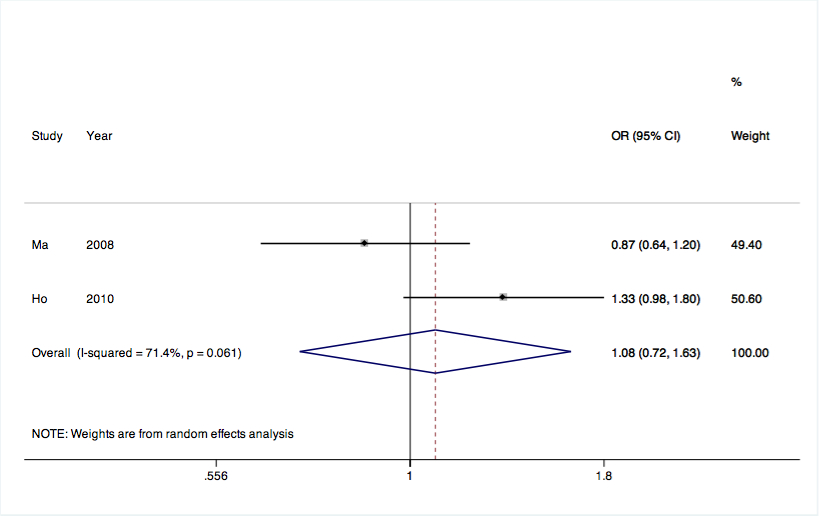


**Supplemental Fig. 5 – Forest plot of studies reporting associations between the rs1695 single nucleotide polymorphism (SNP) of the glutathione S-transferase pi 1 gene and lower urinary tract symptoms. RefSNP alleles A/G. Plot presented as risk associated with minor G allele.**


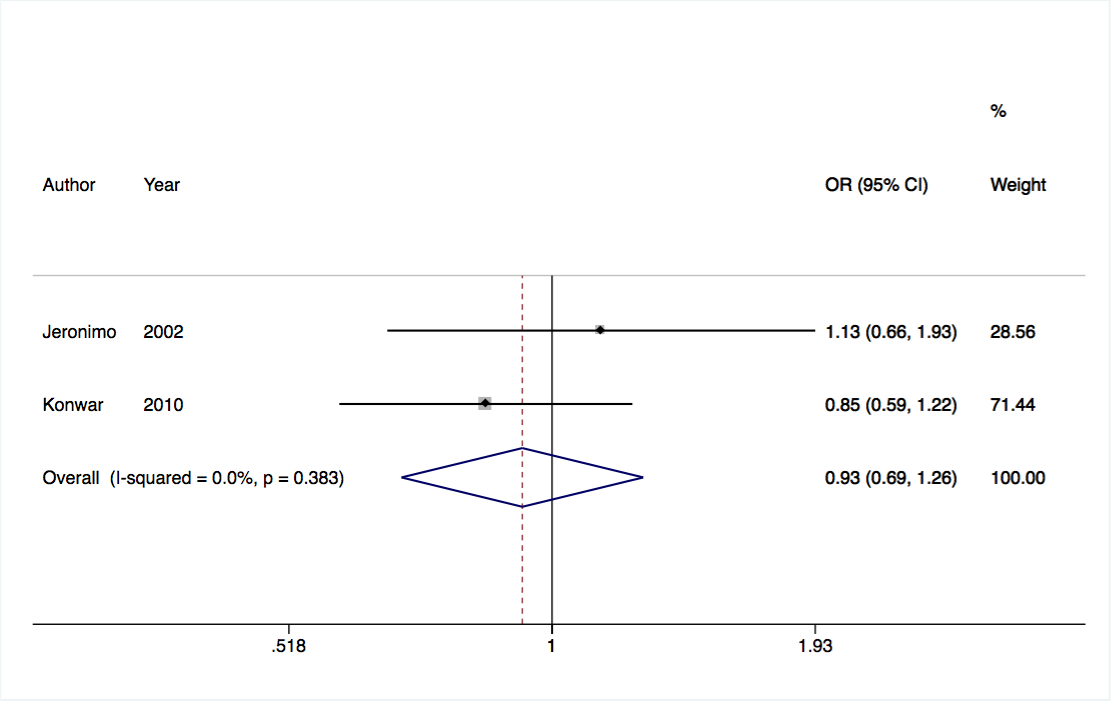


**Supplemental Fig. 6 – Forest plot of studies reporting associations between the null allele of the glutathione S-transferase theta 1 gene and lower urinary tract symptoms. Plot presented as risk associated with deletion/null allele.**


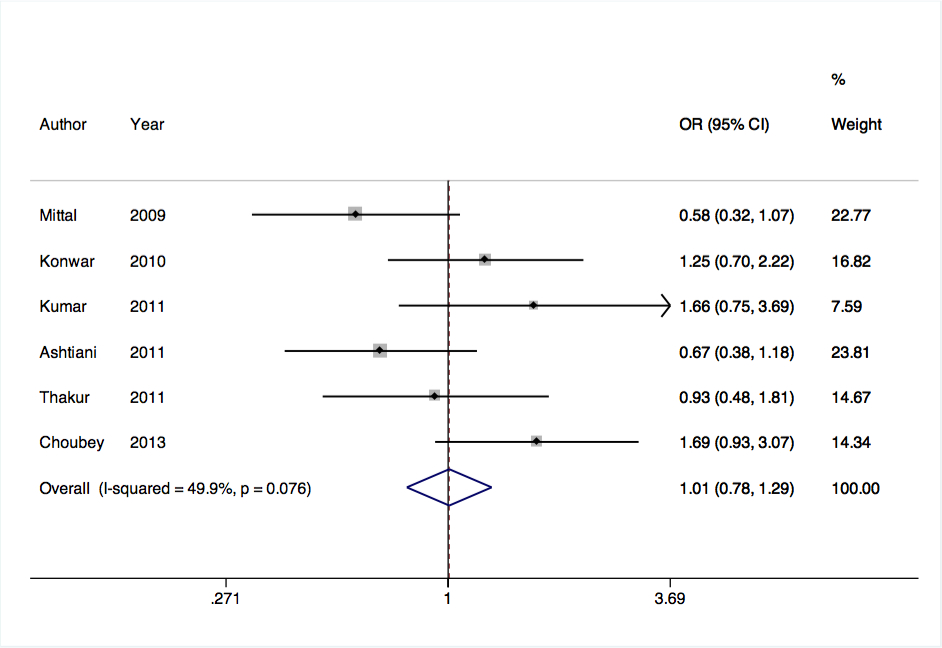


**Supplemental Fig. 7 – Forest plot of studies reporting associations between the rs2430561 single nucleotide polymorphism (SNP) of the interferon gamma gene and LUTS. RefSNP alleles A/T. Plot presented as risk associated with minor A allele.**


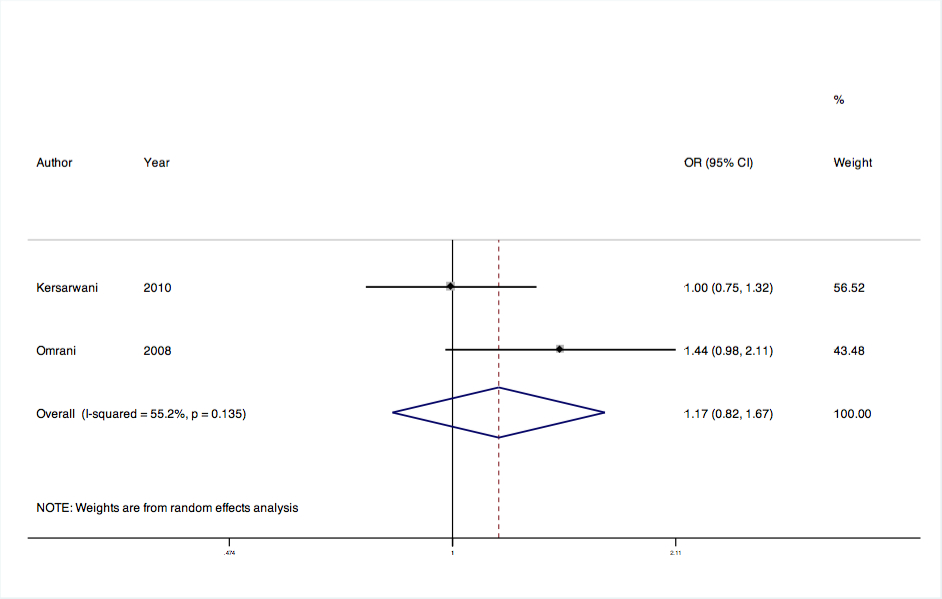


**Supplemental Fig. 8 – Forest plot of studies reporting associations between the rs2854744 single nucleotide polymorphism (SNP) of the insulinlike growth factor-binding protein 3 gene and lower urinary tract symptoms. RefSNP alleles A/C. Plot presented as risk associated with minor C allele.**


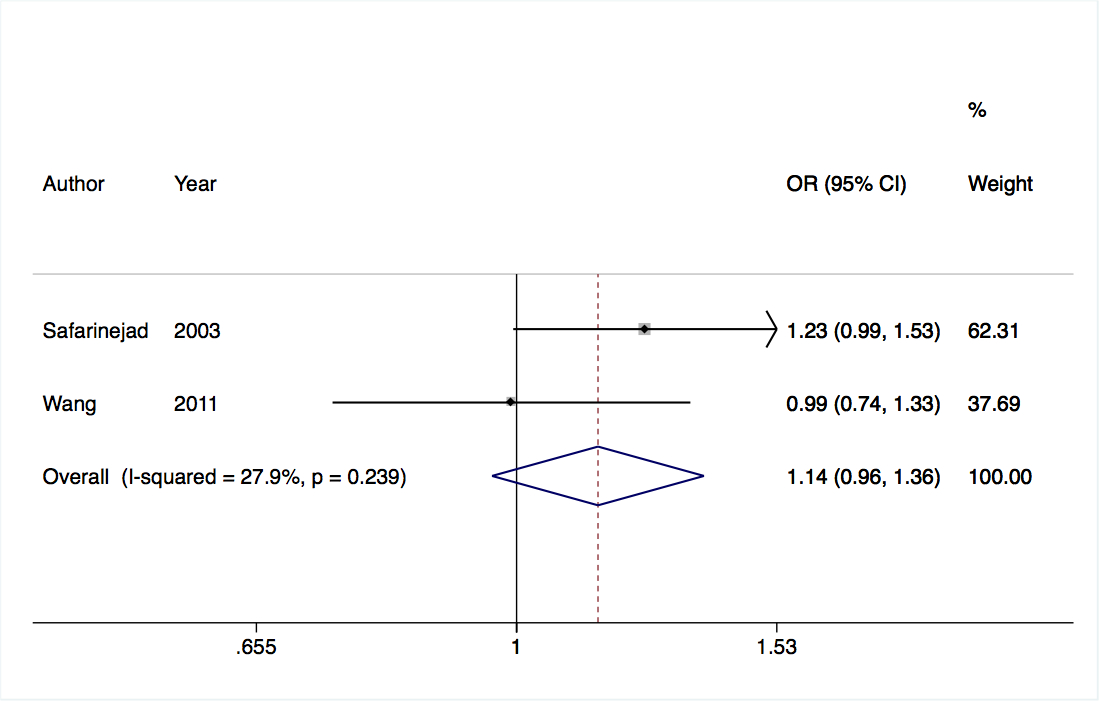


**Supplemental Fig. 9 – Forest plot of studies reporting associations between the rs1800896 single nucleotide polymorphism (SNP) of the interleukin 10 gene and lower urinary tract symptoms. RefSNP alleles A/G. Plot presented as risk associated with minor G allele.**


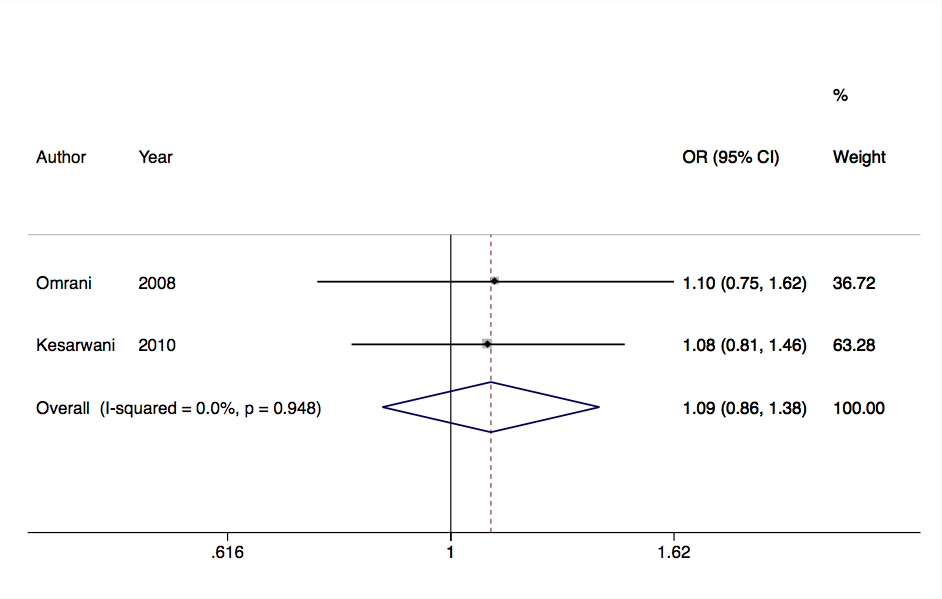


**Supplemental Fig. 10 – Forest plot of studies reporting associations between the rs2234663 short tandem repeat of the interleukin 1 receptor antagonist gene and lower urinary tract symptoms. Plot presented as risk associated with 410bp allele.**


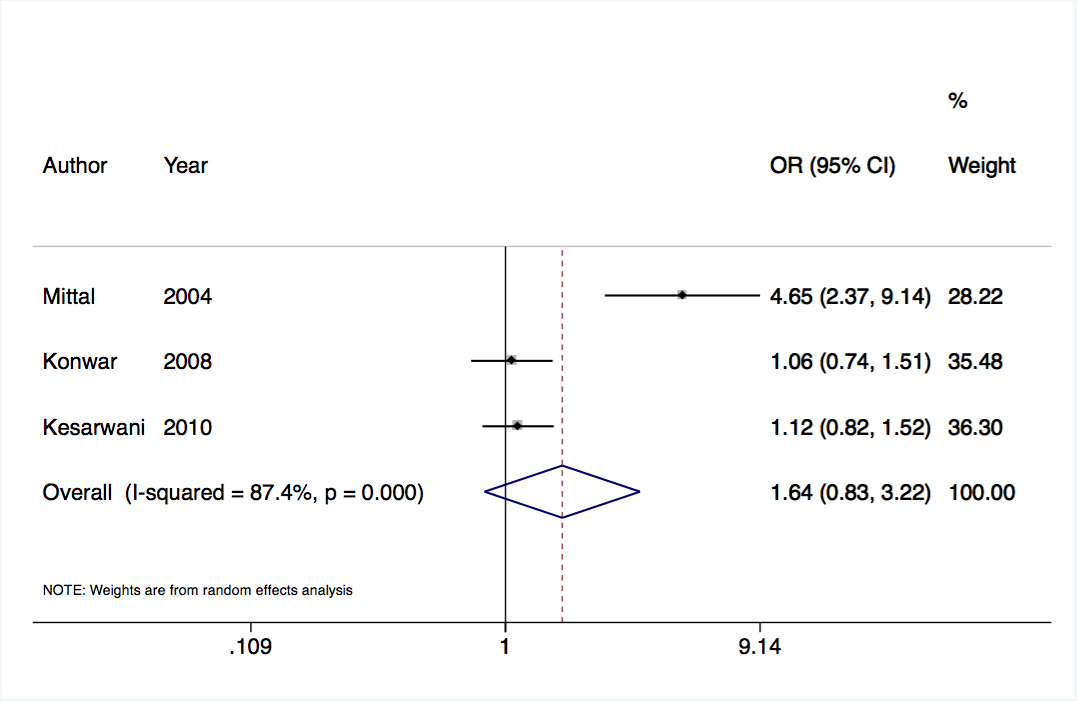


**Supplemental Fig. 11 – Forest plot of studies reporting associations between the rs2234664 INDEL of the interleukin 4 gene and lower urinary tract symptoms. RefSNP alleles A/G. Plot presented as risk associated with three copies (relative to two copies).**


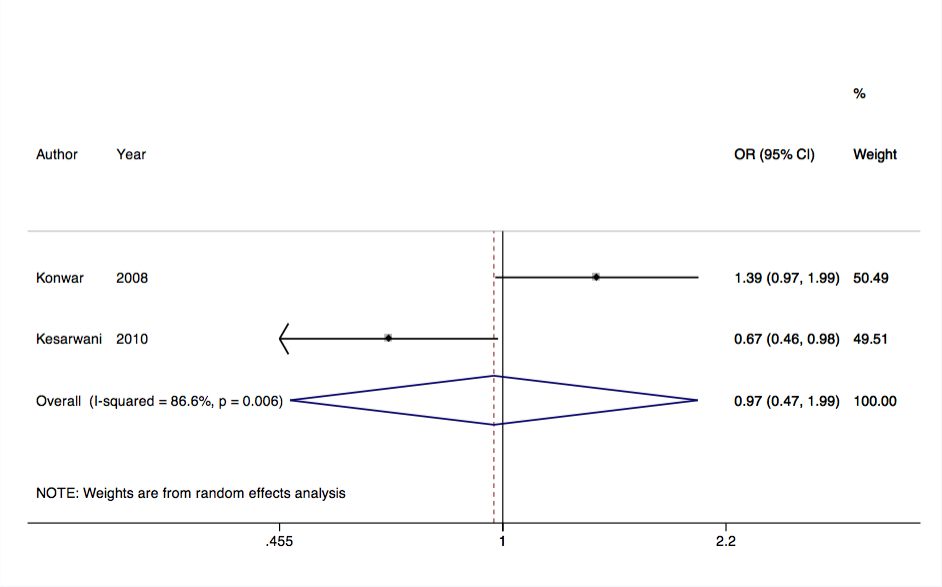


**Supplemental Fig. 12 – Forest plot of studies reporting associations between the rs266882 SNP of the kallikrein-related peptidase 3 gene and LUTS. RefSNP alleles A/G. Plot presented as risk associated with minor A allele.**

Note: Forest plots for rs17632542 and rs2735839 polymorphisms of the same gene shown in Figure 6.


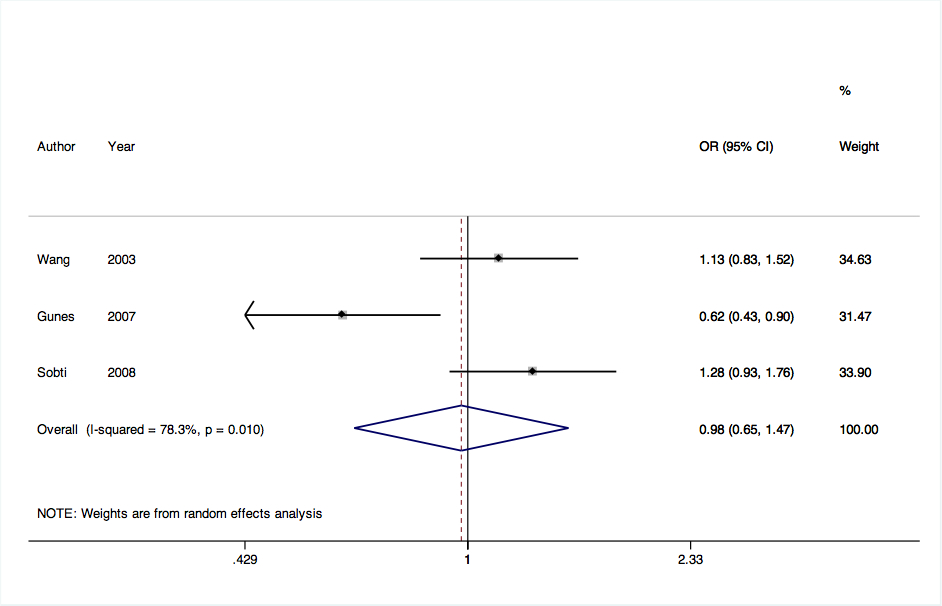


**Supplemental Fig. 13 – Forest plot of studies reporting associations between the rs1447295 and rs6983267 single nucleotide polymorphisms of the RP11-38 gene and lower urinary tract symptoms. Plots presented as risk associated with minor alleles.**


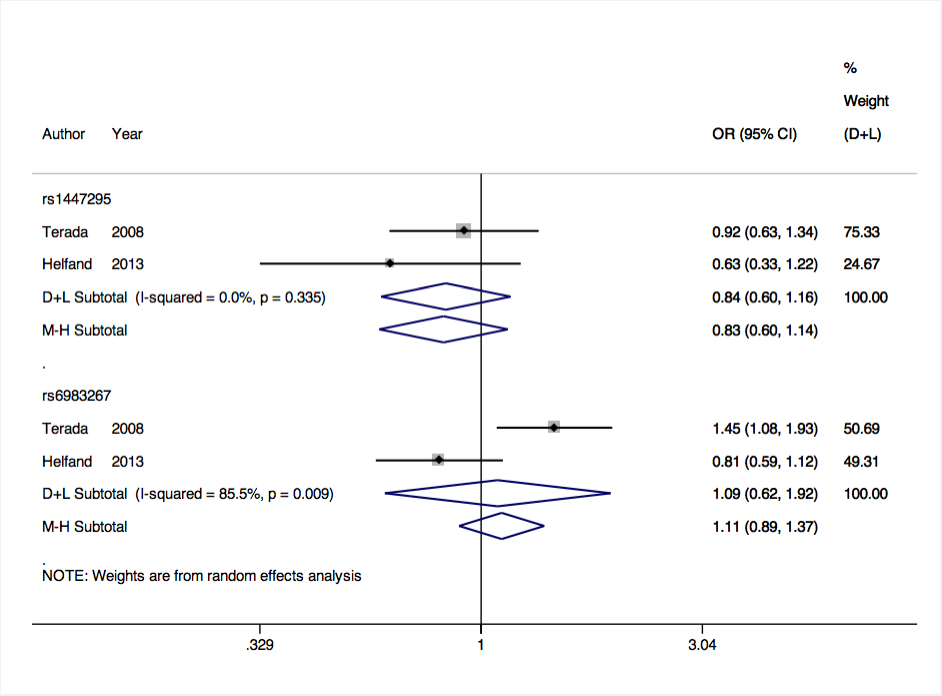


**Supplemental Fig. 14 – Forest plot of studies reporting associations between the rs523349, rs9282858, and TA(n) polymorphisms of the steroid-5α-reductase, α polypeptide 2 gene, and lower urinary tract symptoms. Plots presented as risk associated with minor alleles for each single nucleotide polymorphism and for nine or more repeats for the TA(n) repeat.**


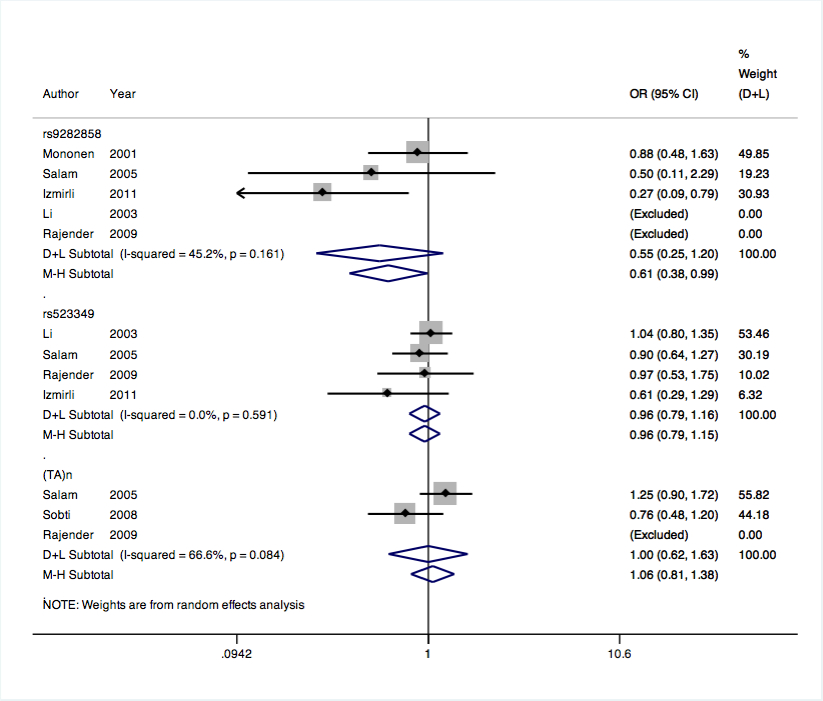


**Supplemental Fig. 15 – Forest plot of studies reporting associations between the rs1800470 single nucleotide polymorphism (SNP) of the transforming growth factor, β 1 gene, and LUTS. RefSNP alleles C/T. Plot presented as risk associated with minor C allele.**


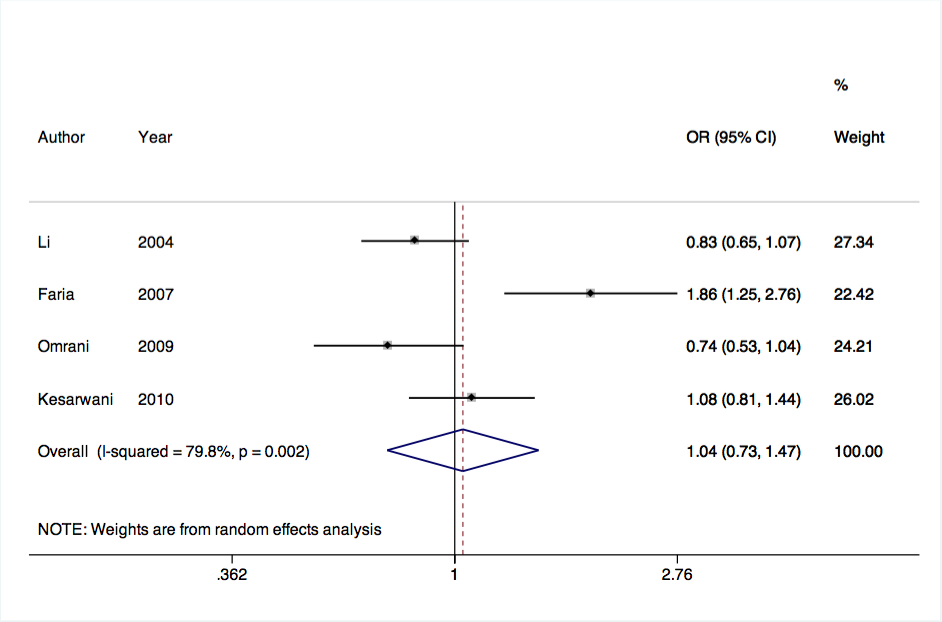


**Supplemental Fig. 16 – Forest plot of studies reporting associations between the rs1800629 single nucleotide polymorphism (SNP) of the tumor necrosis factor gene and lower urinary tract symptoms. RefSNP alleles A/G. Plot presented as risk associated with minor A allele.**


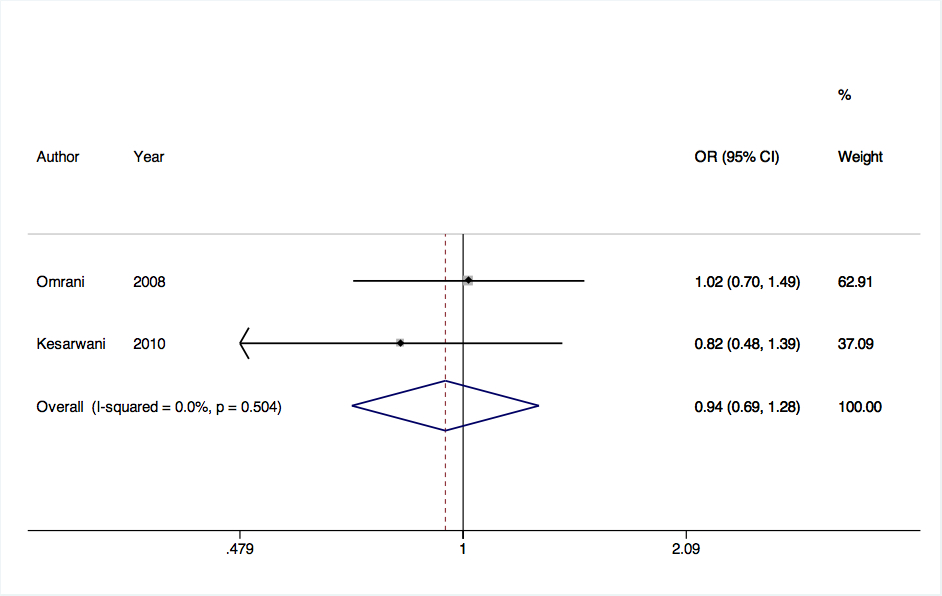


**Supplemental Fig. 17 – Summary of interim Venice guideline ratings of credibility of genetic associations. Strong credibility for an association requires AAA rating. Any B rating confers maximum moderate credibility; any C rating confers weak credibility. Abridged from Table 3 in Ioannidis et al [22].**

| Criteria | Categories |
| --- | --- |
| Amount of evidence | **A**: Large-scale evidence  (n>1000 with risk allele)  **B**: Moderate amount of evidence (n 100-1000)  **C**: Little evidence (n<100) |
| Replication | **A**: Extensive replication including at least one well-conducted meta-analysis with little between-study inconsistency (I^2^<25%)  **B**: Well-conducted meta-analysis with some methodological limitations or moderate between-study inconsistency (I^2^ 25%-50%)  **C**: No association; no independent replication; failed replication; scattered studies; flawed meta-analysis or large inconsistency (I^2^>50%) |
| Protection from bias | **A**: Bias, if at all present, could affect the magnitude but probably not the presence of the association  **B**: No obvious bias that may affect the presence of the association but there is considerable missing information on the generation of evidence  **C**: Considerable potential for or demonstrable bias that can affect even the presence or absence of the association |
